# Nitrogen-fixing symbiosis induces differential accumulation of *Medicago truncatula* leaf defence metabolites in response to pea aphid infestation

**DOI:** 10.3389/fpls.2025.1670344

**Published:** 2025-11-20

**Authors:** Goodluck Benjamin, Marie Pacoud, Stéphanie Boutet, Gilles Clement, Renaud Brouquisse, Jean-Luc Gatti, Marylène Poirié, Pierre Frendo

**Affiliations:** 1 Université Côte d’Azur, Institut National de Recherche pour l'Agriculture, l'Alimentation et l'Environnement (INRAE), Centre National de la Recherche Scientifique (CNRS), Institut Sophia Agrobiotech, Sophia Antipolis, France; 2 Université Paris-Saclay, INRAE, AgroParisTech, Institut Jean-Pierre Bourgin for Plant Sciences (IJPB), Versailles, France

**Keywords:** *Medicago truncatula*, *Sinorhizobium meliloti*, nitrogen-fixing symbiosis, *Acyrthosiphon pisum*, metabolomics, plant defence priming

## Abstract

Legume symbiosis with rhizobial nitrogen-fixing bacteria enables legumes to grow in nitrate-depleted soils. Rhizobial symbioses also induce systemic plant defence against bioaggressors. We investigated how nitrogen-fixing symbiosis (NFS) in the legume *Medicago truncatula* can prime plant defence against the pea aphid *Acyrthosiphon pisum*. We analysed metabolite modification using both gas chromatography/mass spectrometry (GC-MS) and liquid chromatography/mass spectrometry (LC-MS) and defence pathway gene expression using qPCR in the leaves of both NFS and nitrate-fed [non-inoculated (NI)] plants after aphid infestation (Amp). The accumulation of primary and secondary metabolites was modulated by both NFS and aphid infestation. Sixty-two defence-related metabolites, such as salicylate, pipecolate, gentisic acid, and several soluble sugars, were differentially regulated by aphid infestation under both NFS and NI conditions. Nineteen metabolites, including triterpenoid saponins, accumulated specifically under NFS_Amp conditions. Gene expression analysis showed that aphid-infested plants exhibited significantly higher expression of *chalcone isomerase*, *flavonol synthase*, *hydroxyisoflavone-O-methyl transferase*, and *pterocarpan synthase*, while *D-pinitol dehydrogenase* was only significantly induced in NI-infested leaves. Our data suggest that NFS, in addition to being a plant nitrogen provider, stimulates specific legume defences upon pest attack and should also be considered a potential tool in Integrated Pest Management strategies.

## Introduction

Plants are under constant threat from pathogens and insect pests such as sap-feeding aphids. Aphids are deleterious agronomic pests, not only because they feed on phloem sap and therefore weaken the plant, but also because they are vectors for various plant viruses (Ng and Perry, 2004). More than 5,000 species of aphids are known today, a diversity that is partly due to sympatric speciation initiated by individuals adapting to new host plants (Diehl and Bush, 1984; Drès and Mallet, 2002). During feeding, the aphid stylet injures plant cells, injects saliva, and sucks up tiny amounts of the cell content to determine plant suitability (Martin et al., 1997; Lu et al., 2016). Previous studies have revealed that the secreted proteins present in saliva trigger plant responses (Pitino and Hogenhout, 2013; Rodriguez and Bos, 2013). These salivary proteins act as herbivore-associated molecular patterns (HAMPs) that bind to host plant pattern recognition receptors (PRRs) and trigger an immune response associated with the production of reactive oxygen species (ROS) and defence hormone salicylic acid (SA) and jasmonic acid (JA) pathways (Kaloshian and Walling, 2016; Wu and Baldwin, 2010; Herrera-Vásquez et al., 2015). The increase in SA and JA regulates the accumulation of various primary and secondary metabolites that play a role in plant defence (Isah, 2019) as feeding deterrents or toxins that decrease food intake or food use efficiency, decrease the survival and reproduction of the pest, or indirectly act as attractants for its natural enemies (War et al., 2012). The secondary metabolites involved in this process include terpenoids, phenolics, cyanogenic glycosides, glucosinolates, and alkaloids (Maag et al., 2015; Züst and Agrawal, 2016). For example, high levels of saponins and phenolic compounds increase aphid mortality and result in reduced pest populations. Flavonoid glycosides also reduce aphid fecundity, while nitrogen-containing compounds cause host plant rejection (Züst and Agrawal, 2016; Kordan et al., 2012).

One property of legume plants is their ability to associate with rhizobia to perform nitrogen-fixing symbiosis (NFS), i.e., the conversion of atmospheric N_2_ into plant-assimilable ammonium. The microbial partner supplies assimilable nitrogen to the plant in exchange for carbon resources and a protective environment in the root nodules (Lee and Hirsch, 2006). NFS has also been reported to potentially provide defence priming to plants against bioaggressors (Benjamin et al., 2022). For example, in pea (*Pisum sativum*), rhizobium inoculation decreased *Didymella pinodes* disease severity and significantly reduced seed infection level (Desalegn et al., 2016; Ranjbar Sistani et al., 2017). NFS has also been reported to modulate resistance to biotrophic pathogens in both *Medicago truncatula* and pea by reducing the penetration and sporulation of the powdery mildew fungus *Erysiphe pisi* (Smigielski et al., 2019). In contrast, studies have shown in soybean that inoculation with rhizobium significantly impacted aphid populations, with pest densities negatively related to the number of root nodules per plant (Brunner et al., 2015). In another case, inoculation with rhizobium increased the reproductive rate of aphids (Dabré et al., 2022), suggesting an effect dependent upon the experimental conditions.

We previously demonstrated that NFS also influences the *M. truncatula*–pea aphid interaction and general plant defence response (Pandharikar et al., 2020). Indeed, a detrimental effect of rhizobia-inoculated plants on aphid development was observed, with lower adult weights compared to aphids from nitrate-fed plants. *Pathogenesis Related 1* (*PR1*) gene expression was upregulated in aphid-infested shoots, indicating the activation of SA-dependent defence. Moreover, a significantly higher expression of the *Proteinase Inhibitor* (*PI*) gene, a marker for the JA transduction pathway, was observed in NFS plants compared to nitrate-fed plants (Pandharikar et al., 2020).

Due to the observations from our preliminary studies, we analysed the impact of nitrogen sources (KNO_3_*vs*. NFS) on the metabolism of *M. truncatula* plants infested with pea aphids. To this end, we analysed the leaf metabolite profiles of NFS plants with and without aphid infestation and compared them to those of KNO_3_-fed plants [non-inoculated (NI)], and to obtain a maximum coverage of the leaf metabolites, we conducted untargeted metabolomics using both gas chromatography/mass spectrometry (GC-MS) and liquid chromatography/mass spectrometry (LC-MS). Our results showed that both primary and secondary metabolism are significantly modulated by nitrogen source and aphid infestation. The gene expression analysis of enzymes involved in the secondary metabolite synthesis pathways showed that the regulation of secondary metabolism is partially mediated by the modulation of gene expression. We identified specific metabolites that are differentially regulated in NFS plants compared to NI plants under aphid infestation.

## Materials and methods

### Biological materials and experimental design

The pea aphid *Acyrthosiphon pisum* clone, YR2-amp, hereinafter referred to as Amp, is derived from a clover biotype line collected in England that was freed from the secondary symbiont *Regiella insecticola* by ampicillin treatment (Simon et al., 2011). This aphid line is stable (more than 15 years old) and was maintained on fava bean, 20 °C, 16:8-h light/dark cycle. To synchronise aphids, 20–40 apterous female adults were placed in a Petri dish containing a fava bean leaf and allowed to reproduce for 24 h. Then, 10 nymphs (L1) were collected and used for infestation.

For each biological replicate, four groups of five pots, each containing six *M. truncatula* A17 plants, were grown as previously described (Pandharikar et al., 2020) (**Supplementary Figure S1**). After 12 days, two groups were inoculated with the nitrogen-fixing bacteria *Sinorhizobium meliloti* 2011 (NFS condition), and two were supplemented once with 10 mL of 5 mM KNO_3_ solution (NI condition). Seven days after (the time to NFS plants to develop nodules), under both NFS and NI conditions, one group of NI plants and one group of NFS plants were infested with aphids (10 L1/pot). The aphid nymphs were then allowed to develop into adults for 12 days. All pots were individually isolated in a ventilated plastic box and maintained at 20°C under a 16:8-h light/dark photoperiod. For each condition, control pots (one group per condition) were treated and maintained under the same conditions except that no aphids were introduced into the plastic box. At the end of the 12 days, harvested leaves were used immediately or frozen in liquid nitrogen and stored at −80°C.

Rhizobium inoculation was conducted using a streptomycin-resistant strain of *S. meliloti* 2011. It was cultured on Luria–Bertani medium supplemented with 2.5 mM CaCl_2_ and MgSO_4_ (LBMC) and streptomycin at 200 μg mL^−1^ for 3 days at 30°C, then transferred and grown in LBMC liquid medium for 24 h, pelleted at 5,000 *g*, washed twice with sterile distilled water, and resuspended in sterile distilled water to a final optical density of 0.05 (OD_600_). Each NFS plant was inoculated with 10 mL of this *S. meliloti* suspension.

A total of four independent biological replicates (i.e., four times) of four groups (NFS_Control, NFS_Amp, NI_Control, and NI_Amp), with five pots per group containing six plants per pot, produced as described above, were used for metabolomic analysis carried out in this study.

### Analysis of metabolites using gas chromatography/mass spectrometry

Leaves were ground in liquid nitrogen to obtain a fine powder, and 50 mg was resuspended in 1 mL of cold (−20°C) water:acetonitrile:isopropanol (2:3:3 in volume) containing 4 μg mL^−1^ ribitol as internal standard. After extraction under shaking (10 min, 4°C), insoluble material was removed by centrifugation at 20,000 *g* for 5 min, and 50 μL was collected and dried overnight (SpeedVac™) and used immediately or stored at −80°C. Three blank tubes underwent the same treatments to estimate possible contamination. A quality control was made by pooling an equal volume of each condition.

Samples were warmed 15 min before opening and dried again for 1.5 h at 35°C before the addition of 10 μL of 20 mg mL^−1^ methoxyamine in pyridine, and the reaction was performed for 90 min at 28°C under continuous shaking; 90 μL of *N*-methyl-*N*-trimethylsilyl trifluoroacetamide (MSTFA) was then added, and the reaction continued for 30 min at 37°C. After cooling, 45 μL was taken for injection; 1 μL of derivatised sample was injected in splitless and split (1:30) modes on a gas chromatograph (Agilent 7890A; Santa Clara, CA, USA) coupled to a mass spectrometer (Agilent 5977B) with a heated separation column (Rxi-5SilMS; Restek, Lisses, France) (temperature ramp: 70°C for 7 min and then 10 °C min^−1^ to 330°C for 5 min; run length 38 min). Helium flow was constant at 0.7 mL min^−1^. Five scans per second were acquired, spanning a range of 50 to 600 Da. The instrument was tuned with Perfluorotributylamine (PFTBA) with *m/z* 69 and *m/z* 219 of equal intensities. Samples were randomised. Three independent quality controls were injected at the beginning, in the middle, and at the end of the analysis for monitoring the derivatisation stability. An alkane mix (C10, C12, C15, C19, C22, C28, C32, and C36) was injected during the run for external calibration. Three independent derivatisations of the quality control were injected at the beginning, in the middle, and at the end of the series. A response coefficient was determined for 4 ng each of a set of 103 metabolites to the same amount of ribitol. This compound was used to give an estimation of the absolute concentration of the metabolite in what we may call a “one-point calibration” (Fiehn, 2006; 2008).

### Analysis of metabolites using liquid chromatography/mass spectrometry

Metabolites were extracted from 6 mg of fresh weight ground sample using a protocol adapted from the literature (Kim et al., 2008). Briefly, 1.6 mL of a mix of methanol/H_2_O/acetone/TFA (40/32/28/0.05, v:v:v:v) and 300 ng of apigenin (used as internal standard) were added to each sample, which was then stirred at 4°C for 30 min. After centrifugation (10 min, 20,000 *g*, 4°C), the supernatant was collected, and the pellet was extracted again by stirring with 1.6 mL of the previous solvent mix for 30 min. After centrifugation, the two supernatants were pooled, dried, and resuspended in 200 μL of water (Ultra-Liquid Chromatography (ULC)/MS grade)/acetonitrile (90/10) (Biosolve Chimie, Dieuze, France) and filtered (filter paper grade GF/A Whatman^®^).

Metabolomic data were acquired using a Ultra-High-Performance Liquid Chromatography (UHPLC) system (Ultimate 3000, Thermo Scientific, Waltham, MA, USA) coupled to a quadrupole time-of-flight mass spectrometer (Q-Tof Impact II Bruker Daltonics, Bremen, Germany). A Nucleoshell RP18 plus reversed-phase column (2 × 100 mm, 2.7 μm; Macherey-Nagel, Hoerdt, France) was used for chromatographic separation. The mobile phases used for the chromatographic separation were (A) 0.1% formic acid in H_2_O and (B) 0.1% formic acid in acetonitrile. The flow rate was 400 μL min^−1^, and the following gradient was used: 95% of A for 1 min, followed by a linear gradient from 95% A to 80% A from 1 to 3 min, then a linear gradient from 80% A to 75% A from 3 to 8 min, and a linear gradient from 75% A to 40% A from 8 to 20 min; 0% of A was held until 24 min, followed by a linear gradient from 0% A to 95% A from 24 to 27 min. Finally, the column was washed with 30% A for 3.5 min and then re-equilibrated for 3.5 min (35-min total run time). Data-dependent acquisition (DDA) methods were used for mass spectrometry data in positive and negative Electrospray ionization (ESI) modes using the following parameters: capillary voltage, 4.5 kV; nebulizer gas flow, 2.1 bar; dry gas flow, 6 L min^−1^; and drying gas in a heated electrospray source temperature, 140°C. Samples were analysed at 8 Hz with a mass range of 100 to 1,500 *m/z*. Stepping acquisition parameters were created to improve the fragmentation profile with a collision radiofrequency (RF) from 200 to 700 Vpp, a transfer time from 20 to 70 µs, and collision energy from 20 to 40 eV. Each cycle included an MS full scan and 5 MS/MS Collision-Induced Dissociation (CID) on the five main ions of the previous MS spectrum.

### Analysis of soluble sugars and starch by enzymatic assays

Soluble sugars (sucrose, glucose, and fructose) were extracted from 150 mg of frozen tissue powder (both roots and leaves separately) with an ethanol and water solution (800 μL at 80% of ethanol) by incubation in a water bath at 80°C for 15 min, with shaking every 5 min. The sample was centrifuged (5 min, 5,000 rpm), and the supernatant was collected. The extraction was repeated using the same conditions with 800 μL of a 50% ethanol solution, 800 μL of 100% water, and then 800 μL 80% ethanol solution. All supernatants were mixed and then evaporated. The dried sample was resuspended in 1 mL of water and kept in the dark at −20°C until soluble sugar analysis.

The residual pellet from sugar extraction was used for starch analysis. Immediately after the removal of the last supernatant, the pellet was resuspended in 3 mL of thermostable α-amylase. The tube was plunged into a boiling water bath and mixed every 2 min. After 6 min, the sample was transferred to a 50°C bath, and 0.1 mL of amyloglucosidase (20 U) was added. The tube was mixed and incubated for 30 min. The entire content of the tube was then transferred to a larger tube, with volume adjusted to 20 mL with distilled water, mixed thoroughly, and centrifuged at 5,000 rpm for 5 min. The clear, undiluted supernatant was used for the determination of glucose released from starch hydrolysis.

Sugar analyses were performed using the Sucrose/D-Fructose/D-Glucose assay kit (Megazyme K-SUFRG; Neogen, Lansing, MI, USA) and the Total Starch HK assay kit (Megazyme K-TSHK) as described by the provider. Spectrophotometric measurements were conducted at 340 nm in cuvettes with a 1-cm optical path.

### Gene expression analysis

For RNA extraction, plant material was ground in liquid nitrogen. Total RNAs from 100 mg of tissue were then isolated using RNAzol^®^ RT (SIGMA, Saint Quentin Fallavier, France), quantified, and analysed on NanoDrop and 1.5% agarose gel electrophoresis to assess the purity. DNA digestion (RQ1 RNase-free DNase) and reverse transcription (GoScript™ Reverse Transcription) were performed as described by the manufacturer (Promega, Madison, WI, USA). qPCR was performed (qPCR Master Mix plus CXR; Promega) using cDNA template and each set of primers. *PR1* (MtrunA17_Chr2g0295371) was used as a SA defence gene marker, and *PI* (PSI-1.2; MtrunA17_Chr4g0014461) was used as a JA pathway activation gene (Pandharikar et al., 2020). Other genes of interest analysed were *chalcone isomerase* (*CHI*; MtrunA17_Chr1g0213011), *flavonol synthase/flavanone 3-hydroxylase* (*FLS/F3H*; MtrunA17_Chr3g0092531), *hydroxyisoflavone-O-methyl transferase* (*HI4′O-MT*; MtrunA17_Chr4g0046341), *D-pinitol dehydrogenase* (*OEPB*; MtrunA17_Chr6g0480011), *phenylalanine ammonia-lyase* (*PAL*; MtrunA17_Chr1g0181091), *pterocarpan synthase* (*PTS*; MtrunA17_Chr7g0259091), and *SAR-DEFICIENT4* (*SARD4*; MtrunA17_Chr1g0202471).

Real-time qPCR was performed with specific primers (10 µM) designed using the NCBI primer design platform (https://www.ncbi.nlm.nih.gov/tools/primer-blast/) as well as from literature (**Supplementary Table S1**), with 1:40 cDNA dilutions using 95°C for 3 min followed by 40 cycles at 95°C for 3 sec and 60°C for 30 sec, and melting curves from 65°C to 95°C in increments of 0.5 °C (AriaMx Real-time PCR machine, Agilent). Cycle threshold values (Ct) were normalised to the average Ct of two housekeeping genes: *MtC27* (MtrunA17_Chr2g0295871) and *a38* (MtrunA17_Chr4g0061551) genes (Del Giudice et al., 2011). The expression of these two genes was not affected by the treatments (**Supplementary Figure S2**). The original Ct obtained (Ariamix software; Agilent) (**Dataset S1**) was further used in the R qPCRBASE package (Hilliou, 2013). For each gene, the expression level of the aphid-infested plants was compared with that of the non-infested control plants. The results of the qPCR analysis were generated from four independent biological repeats.

### Metabolomic data processing

For GC-MS, raw data files were converted into the NetCDF format and analysed using the AMDIS software (http://chemdata.nist.gov/mass-spc/amdis/). A home retention index/mass spectral library built from the National Institute of Standards and Technology (NIST) (https://webbook.nist.gov/chemistry/), Golm (http://gmd.mpimp-golm.mpg.de/), and Fiehn databases (https://fiehnlab.ucdavis.edu), and standard compounds were used for metabolite identification. Peak areas were also determined using the TargetLynx software (Waters, Saint-Quentin-en-Yveline, France) after the conversion of the NetCDF file into MassLynx format. AMDIS and TargetLynx in splitless and split 30 modes were compiled in a single Excel file for comparison. After blank mean subtraction, peak areas were normalised to ribitol and leaf fresh weight.

For LC-MS, data files were converted and treated as previously described (Boutet et al., 2022). A first search was conducted in library using the open-source software MZmine2 (Pluskal et al., 2010) with an identification module and “custom database search” to begin the annotation with our library, currently containing 159 annotations (reverse transcription (RT) and *m/z*) in positive mode and 61 in negative mode, with RT tolerance of 0.3 min and *m/z* tolerance of 0.0025 Da or 6 ppm. Molecular networks were generated with the MetGem software (Olivon et al., 2018) (https://metgem.github.io) using the.mgf and.csv files obtained via MZmine2 analysis. ESI− and ESI+ molecular networks were generated using cosine score thresholds of 0.8 in two modes. Metabolite annotation was performed as described (Boutet et al., 2022).

### Statistical analysis

Statistical analysis was performed using TMEV (https://sourceforge.net/projects/mev-tm4/) for GC-MS. Univariate analyses by permutation (one-way ANOVA) were first used to select the significant metabolites (*p*-value < 0.01). Multivariate analyses (hierarchical clustering and principal component analysis) were then made on both LC-MS and GC-MS data. The significant compounds were used for further analysis to identify statistical differences between the different conditions for both primary and secondary metabolites by Tukey’s multiple comparison tests performed on independent metabolites. This statistical analysis of individual metabolites was conducted using Prism v9.1.1 (GraphPad Software, USA). All experimental data are expressed as mean ± standard error (SE). A Venn diagram was created based on the results of Tukey’s tests, clustering metabolites based on statistical significance (*p*-value ≤ 0.05) using InteractiVenn (Heberle et al., 2015).

## Results

### Aphid infestation significantly alters leaf metabolite profile

**Figure 1:**
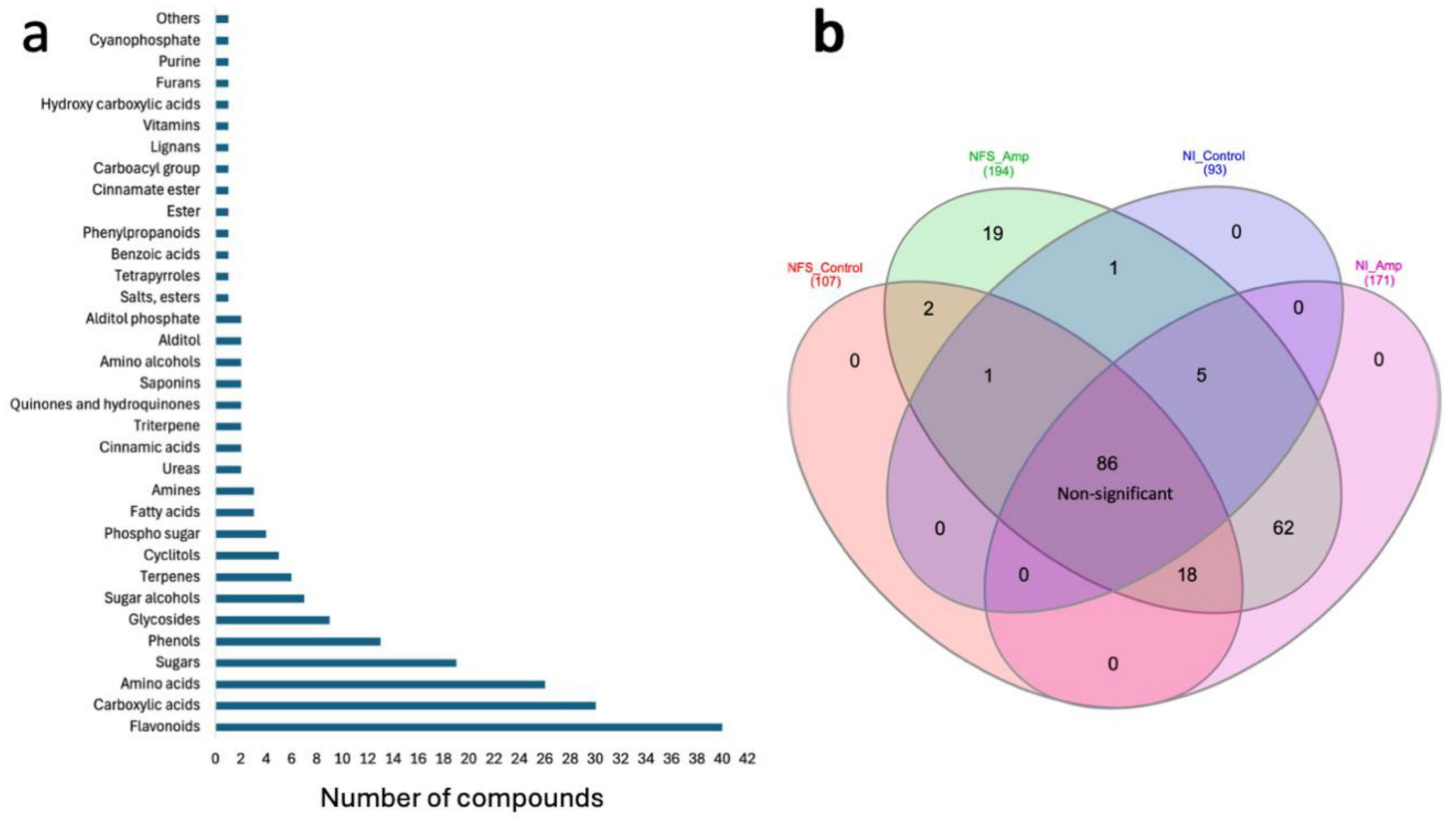
**(a)** Aphid infestation, more than nitrogen source, significantly alters leaf metabolite profile. Class abundance of metabolites from both GC-MS and LC-MS analyses. **(b)** Metabolite distribution according to nitrogen source and aphid infestation affects the metabolite profile of plants. Venn diagram showing distribution of statistically significant metabolites across the various experimental conditions. The names of the compounds and the statistics can be found in **Dataset S3** (NFS, nitrogen-fixing symbiosis; NI, non-inoculated; Amp, aphid infestation; control, no aphid infestation).

*M. truncatula* leaf metabolites were analysed under four experimental conditions: nitrate-fed plants (NI), nitrate-fed plants infested with aphids (NI_Amp), nitrogen-fixing symbiotic plants (NFS), and nitrogen-fixing symbiotic plants infested with aphids (NFS_Amp) (**Supplementary Figure S3**). The leaf extracts were analysed using GC-MS to obtain mainly primary metabolites and using LC-MS to have access to the secondary metabolites. After GC-MS, 237 compounds were retained, and 126 could be identified with confidence (**Dataset S2**); amongst them, the five main quantitative compounds were sucrose, phosphate, malate, citrate, and glutamate (**Supplementary Figure S4**). Using LC-MS, 2,627 compounds (in positive and negative modes) were obtained, and 213 could be identified (**Dataset S2**). A strong effect of aphid infestation and nitrogen source on the accumulation of metabolites was observed, and the hierarchical clustering of these compounds separated both nitrogen source and plant infestation status (**Dataset S3**). From the four experimental conditions, a total of 194 unique metabolites, 126 from GC-MS and 69 from LC-MS, were found significantly different between conditions according to their accumulation (**Dataset S2**). Amongst the 194 identified, the most represented classes were flavonoids with 40 different compounds, followed by 30 carboxylic acids, 26 amino acids, and 19 sugars (**Figure 1a**).

To test the effect of the NFS on the plant defence against aphids, we used a multiple comparison test to identify the statistical differences for these 194 unique metabolites between the four experimental conditions (**Figure 1b**; **Dataset S3**). In this analysis, 86 of these compounds were not found to be significantly accumulated under one specific condition, including many of the amino acids. One hundred eight compounds were found to be differentially accumulated under the different conditions. Amongst them, aphid infestation significantly increased the accumulation of 62 metabolites. At least 18 metabolites accumulated under control NFS conditions and NI_Amp conditions, suggesting that they are linked to a general infection/infestation response. Two compounds (5-hydroxynorvaline and tartrate) were specifically found under NFS conditions, and 19 compounds were significantly accumulated under NFS_Amp conditions.

### Aphid infestation triggers a greater accumulation of sugars than amino acids in both NFS and NI plants

#### Analysis of amino acid accumulation

The majority of the amino acids (20) were not significantly accumulated under any specific condition. In contrast, tryptophan, cysteine, and asparagine were significantly accumulated under NFS control, NFS_Amp, and NI_Amp (**Table 1**; **Dataset S3**).

**Table 1:** Amino acid accumulation under the different treatment conditions.

Clusters	Amino acids	NFS_Control	NFS_Amp	NI_Control	NI_Amp	*p*-Value	F	R^2^
[NFS_Control] and [NFS_Amp] and [NI_Control] and [NI_Amp]	Alanine	(81.29 ± 26.90) × 10^−4^	(104.70 ± 32.30) × 10^−4^	(56.92 ± 15.40) × 10^−4^	(92.05 ± 16.50) × 10^−4^	0.5575	0.7228	0.1531
	Arginine	(18.14 ± 1.45) × 10^−4^	(18.66 ± 1.59) × 10^−4^	(16.69 ± 11.00) × 10^−4^	(13.63 ± 4.79) × 10^−4^	0.9362	0.1367	0.03304
	Aspartate	(950.40 ± 53.50) × 10^−4^	(819.90 ± 20.20) × 10^−4^	(616.70 ± 165.00) × 10^−4^	(811.20 ± 234.00) × 10^−4^	0.4756	0.8869	0.1815
	Beta-alanine	(6.58 ± 0.79) × 10^−4^	(6.49 ± 0.64) × 10^−4^	(5.298 ± 1.55) × 10^−4^	(6.90 ± 2.00) × 10^−4^	0.8483	0.2665	0.06246
	Glutamate	(2,867 ± 189) × 10^−4^	(2,957 ± 142) × 10^−4^	(2,329 ± 511) × 10^−4^	(2,983 ± 682) × 10^−4^	0.7003	0.483	0.1077
	Glutamine	(264.10 ± 34.20) × 10^−4^	(194.80 ± 17.10) × 10^−4^	(137.40 ± 66.10) × 10^−4^	(151.1 ± 42.30) × 10^−4^	0.2184	1.707	0.2991
	Glycine	(8.610 ± 1.13) × 10^−4^	(8.15 ± 0.64) × 10^−4^	(9.97 ± 4.59) × 10^−4^	(9.06 ± 2.81) × 10^−4^	0.9705	0.07837	0.01922
	Histidine	(9.056 ± 0.36) × 10^−4^	(16.54 ± 2.20) × 10^−4^	(4.12 ± 2.71) × 10^−4^	(12.89 ± 5.53) × 10^−4^	0.0974	2.638	0.3974
	Homoserine	(7.42 ± 1.44) × 10^−4^	(8.414 ± 2.39) × 10^−4^	(3.374 ± 1.70) × 10^−4^	(2.604 ± 0.65) × 10^−4^	0.0724	3.006	0.4291
	Isoleucine	(25.15 ± 2.43) × 10^−4^	(37.99 ± 7.38) × 10^−4^	(24.11 ± 4.53) × 10^−4^	(47.15 ± 9.12) × 10^−4^	0.0752	2.958	0.4251
	Leucine	(33.39 ± 3.77) × 10^−4^	(42.56 ± 8.91) × 10^−4^	(32.67 ± 6.22) × 10^−4^	(57.88 ± 10.70) × 10^−4^	0.1372	2.231	0.358
	Lysine	(14.58 ± 1.67) × 10^−4^	(13.75 ± 1.52) × 10^−4^	(9.732 ± 3.01) × 10^−4^	(16.88 ± 6.23) × 10^−4^	0.5905	0.663	0.1422
	Methionine	(36.88 ± 1.60) × 10^−4^	(51.24 ± 5.38) × 10^−4^	(30.50 ± 10.60) × 10^−4^	(53.26 ± 9.61) × 10^−4^	0.1565	2.08	0.3421
	Phenylalanine	(24.29 ± 1.51) × 10^−4^	(36.01 ± 4.19) × 10^−4^	(21.03 ± 34.5) × 10^−4^	(38.79 ± 8.14) × 10^−4^	0.0682	3.083	0.4353
	Proline	(64.29 ± 7.71) × 10^−4^	(83.68 ± 8.26) × 10^−4^	(51.12 ± 18.5) × 10^−4^	(108.5 ± 22.50) × 10^−4^	0.1046	2.551	0.3894
	Serine	(172.60 ± 20.40) × 10^−4^	(279.20 ± 18.70) × 10^−4^	(177.00 ± 61.30) × 10^−4^	(299.00 ± 66.30) × 10^−4^	0.1701	1.985	0.3316
	Threonine	(110.10 ± 5.92) × 10^−4^	(127.6 ± 5.55) × 10^−4^	(85.07 ± 26.10) × 10^−4^	(126.9 ± 27.90) × 10^−4^	0.4088	1.043	0.2068
	Tyrosine	(20.50 ± 2.29) × 10^−4^	(28.26 ± 2.75) × 10^−4^	(18.02 ± 2.53) × 10^−4^	(33.37 ± 8.78) × 10^−4^	0.1579	2.069	0.3409
	Valine	(50.99 ± 2.66) × 10^−4^	(75.70 ± 12.00) × 10^−4^	(43.78 ± 9.17) × 10^−4^	(81.24 ± 15.60) × 10^−4^	0.0849	2.807	0.4124
	l-Abrine	21.48 ± 2.12	40.30 ± 5.49	24.63 ± 11.02	48.18 ± 7.01	0.0648	3.149	0.4405
[NFS_Control] and [NFS_Amp]	5-Hydroxynorvaline	(5.99 ± 0.78) × 10^−4^	(5.81 ± 0.41) × 10^−4^	(2.47 ± 0.96) × 10^−4^	(2.83 ± 0.35) × 10^−4^	0.0037	7.803	0.6611
[NFS_Control] and [NFS_Amp] and [NI_Amp]	Asparagine	(4,006 ± 801) × 10^−4^	(3,510 ± 627) × 10^−4^	(806 ± 637) × 10^−4^	(1,537 ± 802) × 10^−4^	0.0241	4.531	0.5311
	Cysteine	(2.96 ± 0.21) × 10^−4^	(3.96 ± 0.07) × 10^−4^	(2.19 ± 0.32) × 10^−4^	(3.52 ± 0.22) × 10^−4^	0.0008	11.41	0.7404
	Tryptophan	(22.95 ± 2.84) × 10^−4^	(58.03 ± 14.60) × 10^−4^	(15.70 ± 2.49) × 10^−4^	(58.83 ± 11.70) × 10^−4^	0.0117	5.688	0.5871

Accumulated amino acids from both LC-MS and GC-MS analyses showing what treatment conditions influence their accumulation. Clusters refer to the Venn diagram from **Figure 1b**. Metabolites analysed and quantified in μg mg^−1^ of fresh weight (FW) of leaves of NFS and NI plants 12 days after infestation by the aphids (NFS, nitrogen-fixing symbiosis; NI, non-inoculated; Amp, aphid infestation; control, no aphid infestation). Mean ± SE (n = 4); *p* ≤ 0.05 (Tukey’s test), significant.

#### Analysis of sugar accumulation

**Figure 2:**
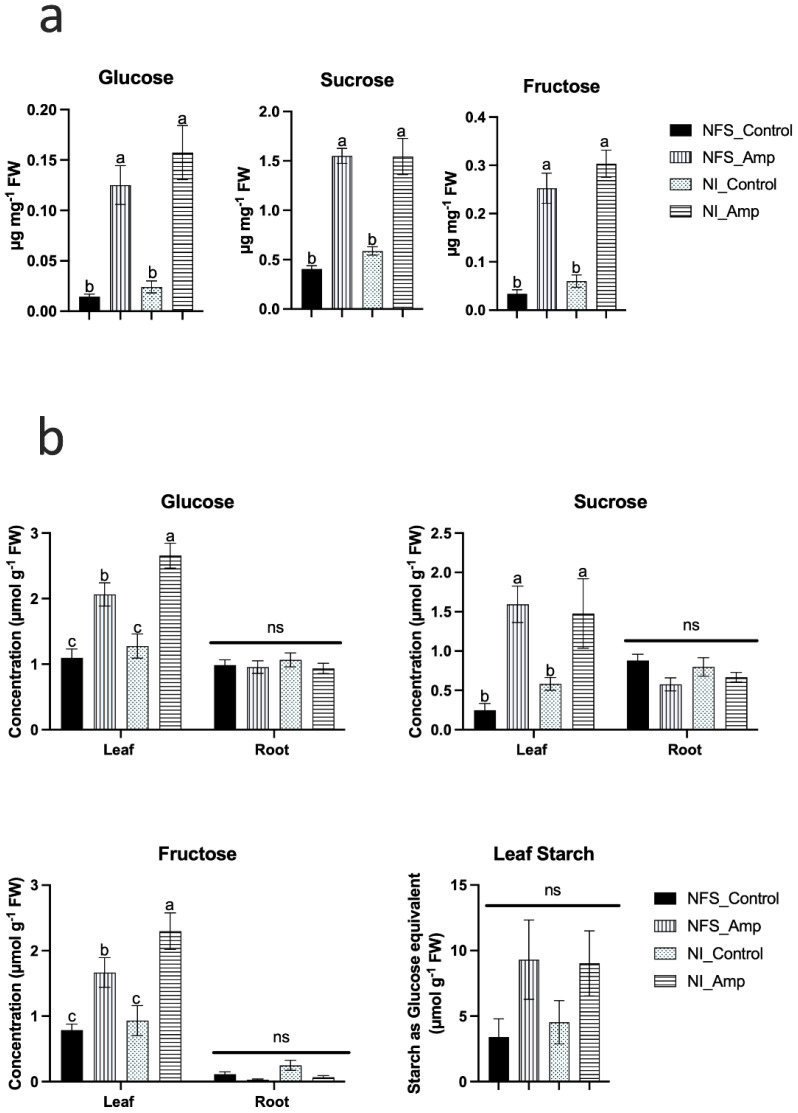
Aphid infestation triggers accumulation of soluble sugars in both NFS and NI plant leaves but not in roots. **a)** Bar graphs from GCMS analysis showing the significant accumulation of glucose sucrose and fructose in NFS and NI plants upon aphid infestation. Metabolites analysed and quantified in μg mg-1 of fresh weight (FW) of leaves of NFS and NI plants 12 days after infestation by the aphids (control = no aphid infestation); **b)** Biochemical analysis of glucose, sucrose, and fructose in leaves showed a significant accumulation in NFS and NI plants under aphid attack. No significant difference was found in roots upon aphid infestation. No statistical difference was observed in leaves starch, measured as glucose equivalent, between the different conditions. Metabolites analysed and quantified in μmol g-1 of fresh weight (FW) of leaves and roots from NFS and NI plants 12 days after infestation by the aphids (NFS, nitrogen-fixing symbiosis; NI, non-inoculated; Amp, aphid infestation; control, no aphid infestation). Mean ± SE (n=4); different letters indicate a p ≤ 0.05 (Tukey’s test).

Amongst the 62 metabolites significantly accumulated under the aphid-infested conditions, glucose, sucrose, and fructose contents were increased between three- and fivefold in leaves compared to those under control conditions (**Figure 2a**). To determine whether this increase in sugar in the leaves was associated with a reduction in sugar transport from leaves to roots or the results of the mobilisation of starch from the leaves, sugar and starch contents were measured using biochemical assays in the leaves and roots of plants under the different conditions (**Figure 2b**). The increase in glucose, sucrose, and fructose in infested leaves was confirmed (**Figure 2b**), but no significant accumulation of starch in leaves and no change in root sugar concentration were observed, regardless of the plant’s growth conditions (**Figure 2b**), suggesting an alteration of the leaf sugar metabolism in response to aphid infestation.

### Aphid infestation triggers accumulation of defence-related metabolites in both NFS and NI plants

**Figure 3:**
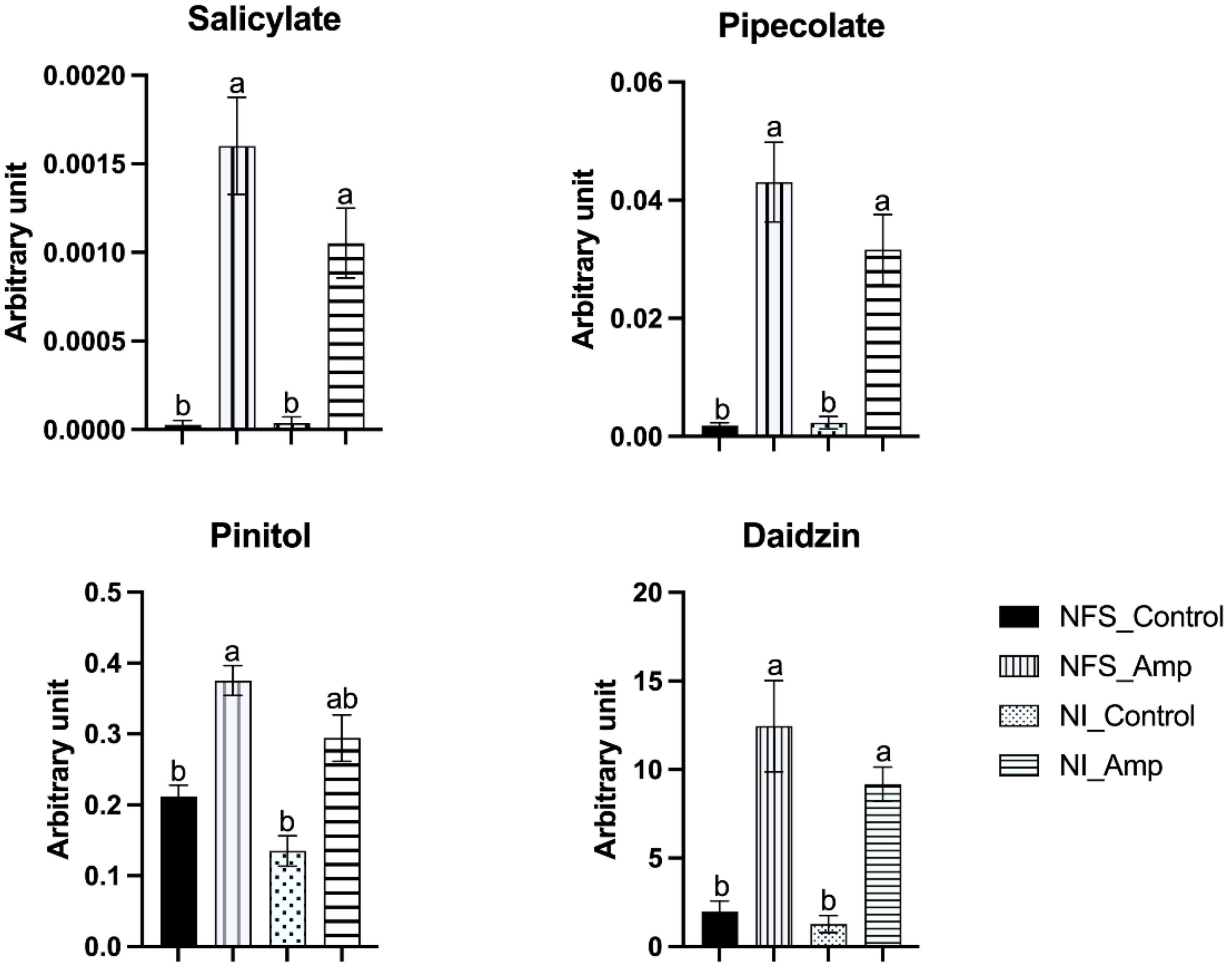
Aphid infestation triggers accumulation of defence-related metabolites in both NFS and NI, particularly from the salicylic acid defence pathway. Graphs showing accumulation of metabolites expressed as arbitrary unit (in mg apigenin equivalent mg^−1^) of leaf fresh weight (FW) of NFS and NI plants 12 days after infestation by the aphids (NFS, nitrogen-fixing symbiosis; NI, non-inoculated; Amp, aphid infestation; control, no aphid infestation). Mean ± SE (n = 4); different letters indicate *p* ≤ 0.05 (Tukey’s test).

Amongst the 62 metabolites significantly accumulated during aphid infestation in both NFS and NI plants (**Figure 1b**; **Dataset S3**), many are involved in the regulation of plant defence pathways such as salicylates, pipecolate (an intermediate of the lysine catabolic pathway), and pinitol (a cyclitol derived from myo-inositol), as well as secondary metabolites with known defence activity such as the putative daidzin, the glycoside form of the aglycone daidzein (**Figure 3**).

### Aphid infestation induces expression of genes involved in the flavonoid synthesis pathway

**Figure 4:**
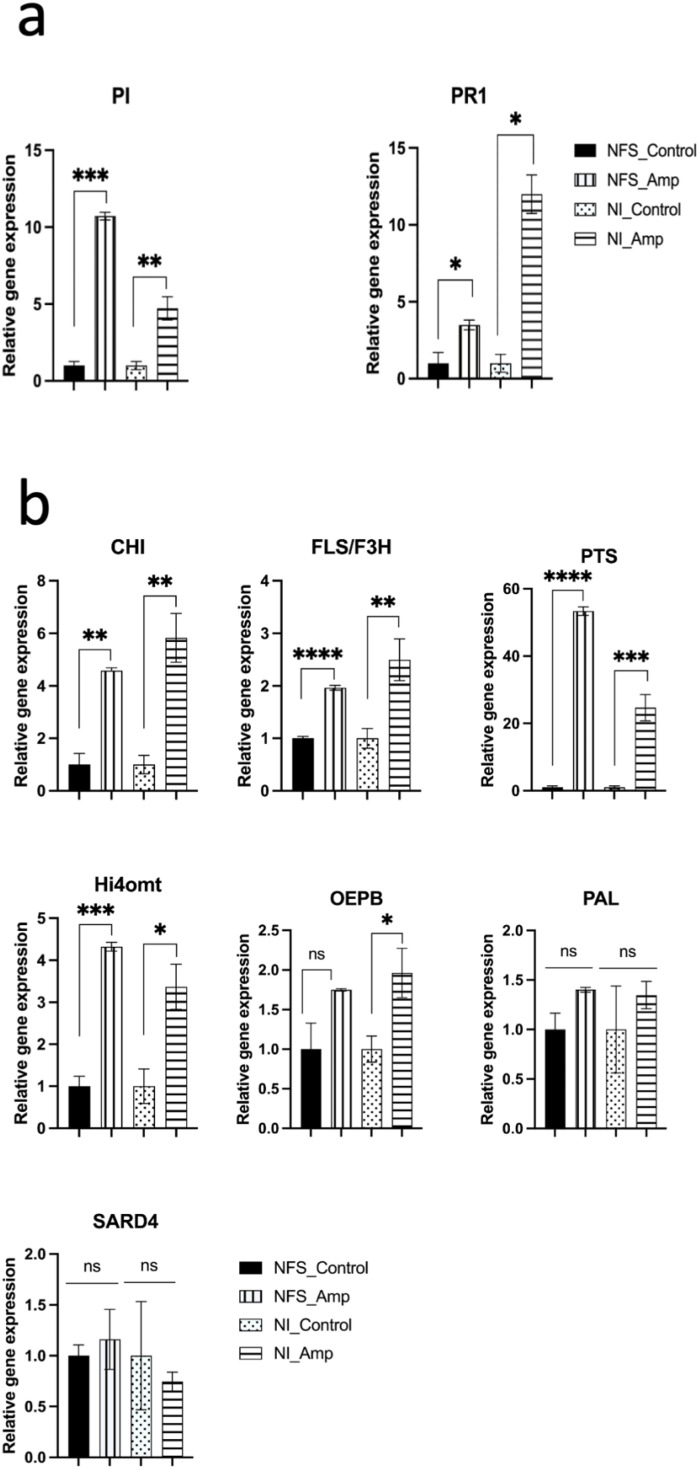
Gene expression analysis of NFS and NI plants. qPCR expression showing the level of gene induction upon aphid infestation in NFS and NI plants (NFS_Amp and NI_Amp, respectively) compared to their control (NFS and NI, respectively) (NFS, nitrogen-fixing symbiosis; NI, non-inoculated; Amp, aphid infestation; control, no aphid infestation). **a)** PR1, a marker for SA defence pathway and PI, a marker for jasmonic acid defence pathway; **b)** genes involved in secondary metabolism pathway (Chalcone isomerase (CHI), flavonol synthase/ flavonone 3β-hydroxylase (FLS/F3H), hydroxyisoflavone-O-methyl transferase (HI4’O-MT) and Pterocarpan synthase (PTS), Phenylalanine Ammonia Lyase (PAL), SAR-DEFICIENT4 (SARD4) and D-pinitol Dehydrogenase (OEPB)). Data are expressed as mean ± standard error (SE); t-test on all genes, p > 0.05, not significant (ns); *, p ≤ 0.05; **, p ≤ 0.01; *** p ≤ 0.001; ****p ≤ 0.0001.

In order to test whether pipecolate, pinitol, and daidzin accumulation resulted also from an increase in the expression of the genes involved in their synthesis pathways, we measured using RT-qPCR the expression of *CHI*, *flavonol synthase/flavonone 3beta-hydroxylase (FLS/F3H)*, *hydroxyisoflavone-O-methyl transferase* (*HI4′O-MT*) and *PTS*, *PAL*, *SAR-DEFICIENT4* (*SARD4*), and *OEPB* (**Supplementary Table S2**). Since we previously showed that *PR1* (a marker for the SA defence pathway) and *PI* (a marker for the jasmonic acid defence pathway) were also differently induced in NFS and NI plants after aphid infestation (Pandharikar et al., 2020), we tested these genes as the plant condition controls. Here, RT-qPCR analysis showed that *PR1* expression increased 3.4 times more under the NI_Amp condition than under the NFS_Amp condition. In contrast, *PI* was 2.3 times more expressed under the NFS_Amp condition than under the NI_Amp condition, in agreement with our previous results (**Figure 4a**; **Supplementary Table S2**). The analysis of the expression of genes involved in the secondary metabolism pathway showed that no significant change in *SARD4* and *PAL* expression was detected in aphid-infested plants compared to the control plants. *OEPB* expression was significantly induced in NI_Amp leaves but not in NFS_Amp leaves, although the amplitude of increase was similar. *CHI*, *FLS/F3H*, *HI4′O-MT*, and *PTS* showed significant induction upon aphid infestation under both NFS and NI conditions, with the induction being twofold for *FLS/F3H* and reaching more than 50-fold for *PTS* in NFS_Amp leaves (**Figure 4b**; **Supplementary Table S2**).

Taken together, these results showed that the accumulation of secondary metabolites is at least partially associated with a higher expression of enzymes involved in their synthesis pathways.

### NFS induces a differential accumulation of defence metabolites upon aphid infestation

**Figure 5:**
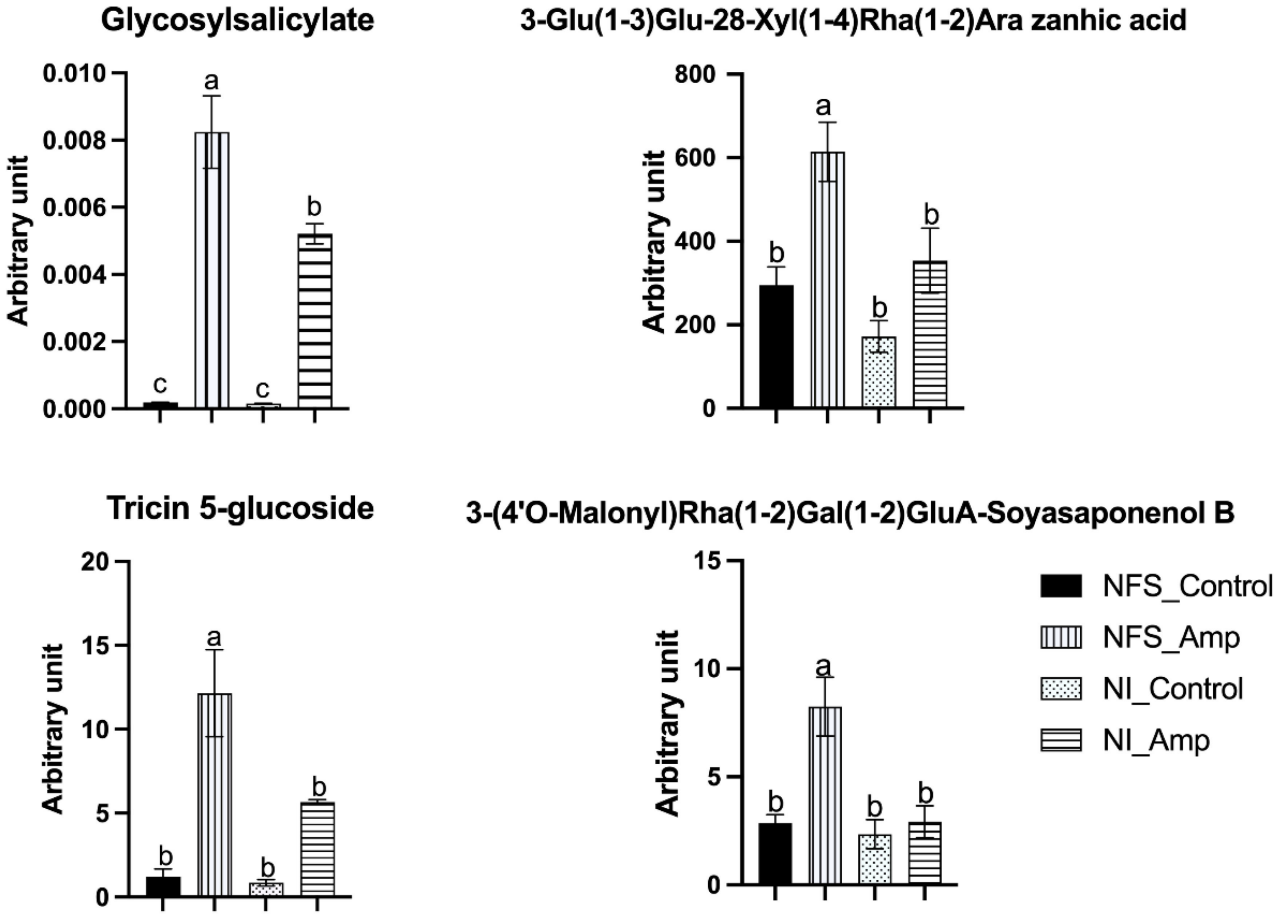
NFS induces a differential accumulation of defence metabolites upon aphid infestation. Graph showing significantly accumulated metabolites in NFS upon aphid infestation. Metabolites expressed as arbitrary unit (in mg apigenin equivalent mg^−1^) of leaf fresh weight (FW) of NFS and NI plants 12 days after infestation by aphids (NFS, nitrogen-fixing symbiosis; NI, non-inoculated; Amp, aphid infestation; control, no aphid infestation). Mean ± SE (n = 4); different letters indicate *p* ≤ 0.05 (Tukey’s test).

Nineteen metabolites (10% of the identified metabolites) were significantly accumulated in NSF_Amp plants compared to other plants (**Dataset S3**). Fifteen of them were secondary metabolites from phenylpropanoid (13) and terpene (two) synthesis pathways. For example, amongst these metabolites, putative triterpenoid saponin 3-Glu(1-3)Glu-28-Xyl(1-4)Rha(1-2)Ara zanhic acid was increased twofold in NFS_Amp plants compared to NI_Amp plants, putative 3-(4′*O*-Malonyl)Rha(1-2)Gal(1-2)GluA-Soyasaponenol B was 2.8 times more accumulated in infested NFS_Amp plants than in the other three plant groups, flavonoid putative tricin 5-glucoside was twice more accumulated in NFS_Amp compared to NI_Amp, and glycosyl salicylate was half a fold more in NFS_Amp plants than in NI_Amp plants (**Figure 5**).

## Discussion

There is a growing interest in a better understanding of the roles of symbiotic microbes in plant defence and, in a broader sense, how this could influence plant interactions with bioaggressors. Beneficial microbes, in addition to enhancing plant growth and development, have been reported to induce defence reactions and confer protection on their host plants (Gopalakrishnan et al., 2015; Liu et al., 2020; Benjamin et al., 2022). Rhizobia are able to induce systemic resistance in legumes such as pigeon pea against *Fusarium* wilt, as it was found that a combination of rhizobia strains was better in inducing resistance (Dutta et al., 2008).

Upon pea infection with the fungus *Didymella*, Turetschek et al. (2017) observed a strong increase in sugars, sugar alcohols, and glycolysis/tricarboxylic acid (TCA) intermediates when studying cultivars in symbiosis with rhizobia and mycorrhiza. They also observed the accumulation of galactose, raffinose, maltose, threitol, melibiose, fructose, and pyruvate in the pea Protecta cultivar. Similar accumulation was also reported for amino acid pools; however, there was significant depletion of phenylalanine in the pea cultivar Messire (Turetschek et al., 2017).

Plants produce diverse primary and secondary metabolites that are involved in various functions, including development and defence. Previous works have demonstrated that aphids are able to modify the overall metabolite profile of plants to establish feeding and that plants may react by producing metabolites that have antifeeding or deterrent effects (Giordanengo et al., 2010; Kumar, 2017; Jakobs et al., 2019; Shih et al., 2023). Few metabolomic studies have focused on pea aphids and legume interactions (Sanchez-Arcos et al., 2019), and none have addressed the role of rhizobia bacteria in plant metabolomic response during aphid attack. Based on our previous observation that nitrogen-fixing symbiosis is detrimental to aphid fitness in *M. truncatula* in association with potential changes in hormonal balance between SA and JA (Pandharikar et al., 2020), we performed an untargeted metabolomic analysis in *M. truncatula* leaves 12 days after aphid infestation (chronic effect) in both NFS and NI plants.

In our study, amongst amino acids (**Dataset S3**), we observed an increase in asparagine content in NFS plant leaves compared to NI control plants, an increase that could be associated with asparagine formation in the root nodule, which accounts for 60% of nodule amino acid content (Sulieman and Schulze, 2010). Similarly, cysteine was found to be more accumulated in symbiotic *Lotus japonicus* plants, as the nodule is an important source of reduced sulphur in the plant (Kalloniati et al., 2015). In contrast, tryptophan accumulation in the leaves of NFS plants has not yet been observed, and a potential role in the defence mechanisms can be assumed (Hiruma et al., 2013). In NFS- and NI-infested plants, the increase in asparagine, cysteine, and tryptophan contents compared to NI plants could be associated with the change of metabolism induced by aphids for better nutritional content (Chiozza et al., 2010; Tegeder, 2014) or by the plant through the induction of defence reaction (Taylor and Ostrowsky, 2019). Citrate, fumarate, malate, and succinate accumulation were not significantly different amongst our four growth conditions, suggesting that the TCA cycle is not changed by NFS or aphid infestation. In contrast to the large number of metabolites that did accumulate differentially under the different growth conditions, two (5-hydroxynorvaline and tartrate) were specifically accumulated under NFS conditions. Whereas the significance of tartrate accumulation, which is an end-point product of the catabolism of ascorbic acid, is more difficult to analyse (Burbidge et al., 2021), unless it is used by the rhizobial bacteria (Ramachandran et al., 2011), that of the 5-hydroxynorvaline, a non-protein amino acid, may be related to its defensive functions against insects (Huang et al., 2011; Yan et al., 2015).

Our data show that the sugar metabolism was particularly affected by aphid infestation (**Figures 2a, b**). Amongst the primary metabolites, 12 were sugars and represented 70% of the number of primary metabolites significantly accumulated in infested plants (**Dataset S3**). In our experimental conditions, we were interested in the sugar transport to the root to feed the root nodule involved in biological nitrogen fixation. Indeed, we previously showed that biological nitrogen fixation is affected under the infested conditions (Pandharikar et al., 2020), and we hypothesised that the decrease in biological nitrogen fixation could be associated with reduced sugar transport from leaves to roots. However, we did not observe a significant modification in the accumulation of root glucose, fructose, and sucrose between control and infested plants, indicating that the modification of sugar metabolism is not associated with the sugar transport to the root. We also observed that starch seemed to be more accumulated in infested plant leaves than in control ones, but this difference was not significant. In conclusion, the modification of sugar metabolism does not seem to be associated with a large modification of sugar storage through starch or sugar transport to roots. Moreover, the modification of sugar metabolism by aphids does not seem to be dependent on the nitrogen source. The accumulation of sugars has been previously reported in plant–aphid (Ponzio et al., 2017) as well as in plant–pathogen interactions (Kanwar and Jha, 2019) as being involved in the coordination of plant defence signalling (Yamada and Mine, 2024). This perhaps could be an explanation for our observation.

A large number of significantly accumulated metabolites (62 metabolites, 31% of the total metabolites) were present in NFS- and NI-infested plants compared to their control counterparts. These results show that *Medicago* plants respond significantly to aphid infestation and that the nitrogen source plays a lesser role in their accumulation. Amongst these secondary metabolites, 72% are from three families: flavonoids (30), phenolics (11), and glycosides (4). These different compounds are mainly associated with plant defence against pests, such as biocide activity (i.e., acacetin, chrysoeriol, and daidzin), feeding deterring activity and defence signalling activity (i.e., salicylate and pipecolate) (Stochmal et al., 2001; Goławska et al., 2012, 2024; Kim et al., 2022; Pawłowska and Stepczyńska, 2022). Pinitol has also been shown to participate in the biological control of powdery mildew in cucumber (Chen et al., 2014), and myo-inositol influences the plant bacterial colonisation (O’Banion et al., 2023). Thus, in parallel with secondary metabolism, the modification of inositol metabolism in the NFS plant may be involved in the differential defence process observed in infested NFS plants compared to infested NI plants. The significant differential accumulation of 3-Glu(1-3)Glu-28-Xyl(1-4)Rha(1-2)Ara zanhic acid and 3-(4′*O*-Malonyl)Rha(1-2)Gal(1-2)GluA-Soyasaponenol B, two terpene molecules are also markers of both the symbiotic state and the infection by aphids. Finally, 3-(4′*O*-Malonyl)Rha(1-2)Gal(1-2)GluA-Soyasaponenol B may be one of the most interesting and intriguing metabolites in our experiment. This triterpenoid saponin is a defensive compound against pathogenic microbes and herbivores and may act as feeding deterrents for plant specialist herbivores (Osbourn, 1996; Kuzina et al., 2009; Szakiel et al., 2011; Cui et al., 2019), causing a cytotoxic effect in the hindgut and fat body of insects (Adel and Sammour, 2012). The production of triterpenoids has also been observed as an effect of rhizobia on pea seeds infected with *Didymella*. The accumulation of seed terpenoid Pisumoside B was observed in uninfected rhizobial-treated seeds; meanwhile, Soyasapogenol C, Api_Dai_Kae_Flavon, and 6-hydroxyapigenin 7-[6″-(3-hydroxy-3-methylglutaryl)glucoside] were significantly enhanced in infected rhizobial-treated seeds in Protecta cultivar (Ranjbar Sistani et al., 2017). 3-(4′*O*-Malonyl)Rha(1-2)Gal(1-2)GluA-Soyasaponenol B may represent a very interesting biological marker of the symbiosis-induced defence priming. The specific accumulation of these defence metabolites affecting the feeding of insects could explain the reduction of the aphid’s fitness that we previously observed under symbiotic conditions (Pandharikar et al., 2020).

The induction of defence mechanisms is associated with the reprogramming of gene expression in plants (Aerts et al., 2022). For example, reciprocal interactions between a chewing herbivore, *Sitona lineatus* (pea leaf weevil), and *P. sativum* (pea) plants grown with or without rhizobia (*Rhizobium leguminosarum* biovar. *viciae*) revealed that plants grown with rhizobia had increased gene transcript expression associated with hormone-related defence (jasmonic acid, ethylene, and abscisic acid) as well as physical and antioxidant-related defence, which may explain the reduced feeding by *S. lineatus* (Basu et al., 2022).

We also looked at the expression of some genes involved in the secondary metabolism pathway. As expected from previous analysis, *PR1* and *PI* were differentially expressed in infested NFS plants and NI plants, suggesting that different defence transcriptional reprogramming occurs under the two conditions. Looking at the genes involved in the secondary metabolism synthesis pathway, the transcript accumulation of *SARD4*, which encodes a key enzyme for pipecolic acid biosynthesis, and *PAL*, the first enzyme of the phenylpropanoid pathway, was not modified by aphid infestation, neither in NFS plants nor in NI plants. This was surprising since *SARD4* was shown to be required for the establishment of systemic acquired resistance to pathogen infection in *Arabidopsis* (Ding et al., 2016). However, *SARD4*-deleted plants were still able to biosynthesise pipecolate; thus, this pathway may involve other enzymes not yet found. PAL is also a member of a multigenic family, and other members of this family may be induced. In contrast, the expression of isoflavonoid pathway genes *CHI*, *FLS/F3H*, *HI4′O-MT*, and *PTS* was significantly increased in infested plants; the induction of *CHI*, *FLS/F3H*, and *HI4′O-MT* did not vary more than twofold under the two infested growth conditions, and the *PTS* expression was induced 53- and 25-fold by aphids in NFS and NI plants, respectively. *PTS* was involved in the synthesis of pterocarpans that constitute the second largest group of natural isoflavonoids and play an important role as phytoalexins. In *Medicago*, the pterocarpan medicarpin was shown to protect the plant from the powdered mildew *E. pisi* and to activate the SA pathway (Gupta et al., 2022). Medicarpin was also shown to be accumulated in *Medicago* leaves upon long-term or strong attack by pea aphids (Stewart et al., 2016) and also in response to infection with the fungal pathogen *Phoma medicaginis* (Jasiński et al., 2009), suggesting some large-spectrum defensive roles. In contrast, medicarpin was shown to be an antagonist of nod gene expression necessary for rhizobia to form their association with the plant roots (Zuanazzi et al., 1998). Thus, an increase in medicarpin synthesis during aphid infestation could also explain in part the effect on the root nodules previously observed (Pandharikar et al., 2020). Surprisingly, *OEPB*, a gene involved in pinitol synthesis (Pupel et al., 2019), was significantly induced in infested NI plants and not in infested NFS plants. Pinitol has been shown to prolong the pea aphid probing behaviour but did not prevent them from feeding (Campbell and Binder, 1984; Kordan et al., 2011). Pinitol has also been involved in the maintenance of the nodule osmotic balance during development, and *S. meliloti* may catabolise pinitol to form nodules (Poole and Ledermann, 2022; Kennedy-Mendez, 2018). Whereas pinitol accumulation was observed in both infested NI plants and infested NFS plants, *OEPB* expression increased only in NI plants, suggesting other regulatory elements associated with the regulation of pinitol.

In conclusion, our results show that under infestation by pea aphids, nitrogen-fixing legumes were able to produce a differential defence reaction by producing specific defence metabolites such as triterpenoid saponins. This specific defence reaction seems to be associated with the JA defence pathway, as the JA-dependent *PI* gene was significantly more expressed under the NFS condition than under the NI condition. One hypothesis is that the defence reaction associated with NFS may be due to differences in plant nitrogen regimes that could directly modulate secondary metabolism. Another hypothesis is that the physical presence of rhizobia within plant cells modulates plant immunity, thereby impacting its subsequent defence against pests. As these hypotheses are not mutually exclusive, further research is necessary to determine the relative importance of each of these factors in the specific defence reaction observed against aphids. Our results provide the foundation for the development of a new form of biocontrol in Integrated Pest Management strategies for legumes. However, multiple questions are still pending to understand the mechanisms underlying our results. Amongst them, the signal pathway associated with this systemic reaction is clear. Indeed, Induced Systemic Resistance (ISR) has already been described in other plants other than legumes. In our biological system, the biological elements associated with defence priming (i.e., intracellular presence of the bacteria, modulated plant defence associated with NFS, and differential nitrogen nutrition) have not been defined. Moreover, the genericity of defence priming is questionable, as we have analysed only one genotype of each partner in this three-way interaction. Nevertheless, our data reinforce and emphasise the results we obtained previously, opening up new avenues for research into the mechanisms underlying defence priming during nitrogen-fixing symbiosis.

## Data Availability

The datasets presented in this study can be found in online repositories. The names of the repository/repositories and accession number(s) can be found in the article/**Supplementary Material**.

## Supplementary material

Supplementary Data Sheet 1 File containing qPCR Data (qPCR CT Data.xlsx).

Supplementary Data Sheet 2 File containing GCMS and LCMS metabolomics data (Metabolomics_data.xlsx).

Supplementary Data Sheet 3 File containing Significant accumulated metabolites analysed by Anova and Venn Analysis (Significant metabolites_Venn.xlsx).

Supplementary Figure 1 Experimental setup. Diagram showing timeline leading up to assay with key time points for bacteria inoculation and aphid infestation marked up. Each pot contained six plants and 5 pots were made for each condition, then 24 plants were used per condition for one biological replicate. Assay was performed using 4 biological replicates produced using the same timeline.

Supplementary Figure 2 Expression of the housekeeping genes used for control in gene expression analysis. Bar graph showing CT of housekeeping genes obtained by RT-qPCR analysis.

Supplementary Figure 3 Experiment treatment classification. Diagram showing the classification of studied treatments into 4 groups, NFS_Control, NFS_Amp, NI_Control (KNO_3_) and NI_Amp (KNO_3_). Amp means aphid infestation condition.

Supplementary Figure 4 Abundance of quantified metabolites. Graph of quantified metabolites from GC-MS analysis showing accuracy of abundance by comparing the mean obtained from the quality controls (QC, blue bars) and mean obtained from the samples (Samples, red bars).

Supplementary Table 1 List of genes and primer sequences used for RT-qPCR analysis.

Supplementary Table 2 Gene expression analysis of NFS and NI plants. Rescaled qPCR expression showing the level of gene induction upon aphid infestation in NFS and NI plants (NFS_Amp and NI_Amp, respectively) compared to their control (NFS and NI, respectively). Data are expressed as mean ± standard error (SE); t-test on all genes, P ≤ 0.05, significant.

## References

[B1] Adel M. M. Sammour E. A. (2012). Effect of sub-lethal dose of natural compound of Medicago sativa (L, Leguminaceae) on the hind gut and fat body of Spodoptera littoralis (Lepidoptera, Noctuidae). J. Appl. Sci. Res. 8, 1398–1408.

[B2] Aerts N. Chhillar H. Ding P. Van Wees S. C. (2022). Transcriptional regulation of plant innate immunity. Essays Biochem. 66, 607–620. doi: 10.1042/EBC20210100, PMID: 35726519 PMC9528082

[B3] Basu S. Lee B. W. Clark R. E. Bera S. Casteel C. L. Crowder D. W. (2022). Legume plant defenses and nutrients mediate indirect interactions between soil rhizobia and chewing herbivores. Basic Appl. Ecol. 64, 57–67. doi: 10.1016/j.baae.2022.08.005

[B4] Benjamin G. Pandharikar G. Frendo P. (2022). Salicylic acid in plant symbioses: Beyond plant pathogen interactions. Biology 11, 861. doi: 10.3390/biology11060861, PMID: 35741382 PMC9220041

[B5] Boutet S. Barreda L. Perreau F. Totozafy J. Mauve C. Gakière B. . (2022). Untargeted metabolomic analyses reveal the diversity and plasticity of the specialized metabolome in seeds of different Camelina sativa genotypes. Plant J. 110, 147–165. doi: 10.1111/tpj.15662, PMID: 34997644

[B6] Brunner S. Goos R. Swenson S. Foster S. Schatz B. Lawley Y. . (2015). Impact of nitrogen fixing and plant growth-promoting bacteria on a phloem-feeding soybean herbivore. Appl. Soil Ecol. 86, 71–81. doi: 10.1016/j.apsoil.2014.10.007

[B7] Burbidge C. A. Ford C. M. Melino V. J. Wong D. C. J. Jia Y. Jenkins C. L. D. . (2021). Biosynthesis and cellular functions of tartaric acid in grapevines. Front. Plant Sci. 12, 643024. doi: 10.3389/fpls.2021.643024, PMID: 33747023 PMC7970118

[B8] Campbell B. C. Binder R. G. (1984). Alfalfa cyclitols in the honeydew of an aphid. Phytochemistry 23, 1786–1787. doi: 10.1016/S0031-9422(00)83492-5

[B9] Chen J. Fernandez D. Wang D. D. Chen Y. J. Dai G. H. (2014). Biological control mechanisms of D-pinitol against powdery mildew in cucumber. Physiol. Mol. Plant Pathol. 88, 52–60. doi: 10.1016/j.pmpp.2014.09.001

[B10] Chiozza M. V. O’Neal M. E. MacIntosh G. C. (2010). Constitutive and induced differential accumulation of amino acid in leaves of susceptible and resistant soybean plants in response to the soybean aphid (Hemiptera: Aphididae). Environ. Entomol 39, 856–864. doi: 10.1603/EN09338, PMID: 20550799

[B11] Cui C. Yang Y. Zhao T. Zou K. Peng C. Cai H. . (2019). Insecticidal activity and insecticidal mechanism of total saponins from Camellia oleifera. Molecules 24, 4518. doi: 10.3390/molecules24244518, PMID: 31835551 PMC6943515

[B12] Dabré É. E. Brodeur J. Hijri M. Favret C. (2022). The effects of an arbuscular mycorrhizal fungus and rhizobium symbioses on soybean aphid mostly fail to propagate to the third trophic level. Microorganisms 10, 1158. doi: 10.3390/microorganisms10061158, PMID: 35744676 PMC9230877

[B13] Del Giudice J. Cam Y. Damiani I. Fung-Chat F. Meilhoc E. Bruand C. . (2011). Nitric oxide is required for an optimal establishment of the Medicago truncatula–Sinorhizobium meliloti symbiosis. New Phytol. 191, 405–417. doi: 10.1111/j.1469-8137.2011.03693.x, PMID: 21457261 PMC3147055

[B14] Desalegn G. Turetschek R. Kaul H.-P. Wienkoop S. (2016). Microbial symbionts affect Pisum sativum proteome and metabolome under Didymella pinodes infection. J. Proteomics 143, 173–187. doi: 10.1016/j.jprot.2016.03.018, PMID: 27016040

[B15] Diehl S. Bush G. (1984). An evolutionary and applied perspective of insect biotypes. Annu. Rev. Entomol 29, 471–504. doi: 10.1146/annurev.en.29.010184.002351

[B16] Ding P. Rekhter D. Ding Y. Feussner K. Busta L. Haroth S. . (2016). Characterization of a pipecolic acid biosynthesis pathway required for systemic acquired resistance. Plant Cell 28, 2603–2615. doi: 10.1105/tpc.16.00486, PMID: 27758894 PMC5134984

[B17] Drès M. Mallet J. (2002). Host races in plant–feeding insects and their importance in sympatric speciation. Philos. Trans. R. Soc. London Ser. B: Biol. Sci. 357, 471–492. doi: 10.1098/rstb.2002.1059, PMID: 12028786 PMC1692958

[B18] Dutta S. Mishra A. Kumar B. D. (2008). Induction of systemic resistance against fusarial wilt in pigeon pea through interaction of plant growth promoting rhizobacteria and rhizobia. Soil Biol. Biochem. 40, 452–461. doi: 10.1016/j.soilbio.2007.09.009

[B19] Fiehn O. (2006). Metabolite Profiling in Arabidopsis. In: Salinas J. Sanchez-Serrano J.J. (eds) Arabidopsis Protocols. Methods in Molecular Biology™, vol 323. Humana Press. doi: 10.1385/1-59745-003-0:439, PMID: 16739598

[B20] Fiehn O. (2008). Extending the breadth of metabolite profiling by gas chromatography coupled to mass spectrometry. TrAC Trends Anal. Chem. 27, 261–269. doi: 10.1016/j.trac.2008.01.007, PMID: 18497891 PMC2394193

[B21] Giordanengo P. Brunissen L. Rusterucci C. Vincent C. Van Bel A. Dinant S. . (2010). Compatible plant-aphid interactions: how aphids manipulate plant responses. Comptes Rendus Biologies 333, 516–523. doi: 10.1016/j.crvi.2010.03.007, PMID: 20541163

[B22] Goławska S. Łukasik I. Chojnacki A. (2024). Luteolin and quercetin affect aphid feeding behavior. Eur. Zoological J. 91, 318–331. doi: 10.1080/24750263.2024.2325544

[B23] Goławska S. Łukasik I. Kapusta I. Janda B. (2012). Do the contents of luteolin, tricin, and chrysoeriol glycosides in alfalfa (Medicago sativa L.) affect the behavior of pea aphid (Acyrthosiphon pisum)? Polish J. Environ. Stud. 21, 1613–1619.

[B24] Gopalakrishnan S. Sathya A. Vijayabharathi R. Varshney R. K. Gowda C. L. Krishnamurthy L. (2015). Plant growth promoting rhizobia: challenges and opportunities. 3 Biotech. 5, 355–377. doi: 10.1007/s13205-014-0241-x, PMID: 28324544 PMC4522733

[B25] Gupta A. Awasthi P. Sharma N. Parveen S. Vats R. P. Singh N. . (2022). Medicarpin confers powdery mildew resistance in Medicago truncatula and activates the salicylic acid signalling pathway. Mol. Plant Pathol. 23, 966–983. doi: 10.1111/mpp.13202, PMID: 35263504 PMC9190973

[B26] Heberle H. Meirelles G. V. Da Silva F. R. Telles G. P. Minghim R. (2015). InteractiVenn: a web-based tool for the analysis of sets through Venn diagrams. BMC Bioinf. 16, 1–7. doi: 10.1186/s12859-015-0611-3, PMID: 25994840 PMC4455604

[B27] Herrera-Vásquez A. Salinas P. Holuigue L. (2015). Salicylic acid and reactive oxygen species interplay in the transcriptional control of defense genes expression. Front. Plant Sci. 6, 131929. doi: 10.3389/fpls.2015.00171, PMID: 25852720 PMC4365548

[B28] Hilliou F. (2013). “ RqPCRAnalysis: analysis of quantitative real-time PCR data,” in Scitepress Lisbon Portugal, 202–211.

[B29] Hiruma K. Fukunaga S. Bednarek P. Piślewska-Bednarek M. Watanabe S. Narusaka Y. . (2013). Glutathione and tryptophan metabolism are required for Arabidopsis immunity during the hypersensitive response to hemibiotrophs. Proc. Natl. Acad. Sci. 110, 9589–9594. doi: 10.1073/pnas.1305745110, PMID: 23696664 PMC3677473

[B30] Huang T. Jander G. De Vos M. (2011). Non-protein amino acids in plant defense against insect herbivores: representative cases and opportunities for further functional analysis. Phytochemistry 72, 1531–1537. doi: 10.1016/j.phytochem.2011.03.019, PMID: 21529857

[B31] Isah T. (2019). Stress and defense responses in plant secondary metabolites production. Biol. Res. 52, 39. doi: 10.1186/s40659-019-0246-3, PMID: 31358053 PMC6661828

[B32] Jakobs R. Schweiger R. Müller C. (2019). Aphid infestation leads to plant part-specific changes in phloem sap chemistry, which may indicate niche construction. New Phytol. 221, 503–514. doi: 10.1111/nph.15335, PMID: 30040116

[B33] Jasiński M. Kachlicki P. Rodziewicz P. Figlerowicz M. Stobiecki M. (2009). Changes in the profile of flavonoid accumulation in Medicago truncatula leaves during infection with fungal pathogen Phoma medicaginis. Plant Physiol. Biochem. 47, 847–853. doi: 10.1016/j.plaphy.2009.05.004, PMID: 19541494

[B34] Kalloniati C. Krompas P. Karalias G. Udvardi M. K. Rennenberg H. Herschbach C. . (2015). Nitrogen-fixing nodules are an important source of reduced sulfur, which triggers global changes in sulfur metabolism in Lotus japonicus. Plant Cell 27, 2384–2400. doi: 10.1105/tpc.15.00108, PMID: 26296963 PMC4815097

[B35] Kaloshian I. Walling LL. (2016). Hemipteran and dipteran pests: Effectors and plant host immune regulators. J. Integr. Plant Biol. 58, 350–361. doi: 10.1111/jipb.12438, PMID: 26467026

[B36] Kanwar P. Jha G. (2019). Alterations in plant sugar metabolism: signatory of pathogen attack. Planta 249, 305–318. doi: 10.1007/s00425-018-3018-3, PMID: 30267150

[B37] Kennedy-Mendez A. I. (2018). Characterization of Pinitol Catabolism in Sinorhizobium Meliloti and its Role in Nodule Occupancy. Kalamazoo Michigan USA, 3417.

[B38] Kim J. H. Cheon Y. M. Kim B.-G. Ahn J.-H. (2008). Analysis of flavonoids and characterization of the OsFNS gene involved in flavone biosynthesis in rice. J. Plant Biol. 51, 97–101. doi: 10.1007/BF03030717

[B39] Kim E.-G. Yun S. Park J.-R. Jang Y.-H. Farooq M. Yun B.-J. . (2022). Bio-efficacy of chrysoeriol7, a natural chemical and repellent, against brown planthopper in rice. Int. J. Mol. Sci. 23, 1540. doi: 10.3390/ijms23031540, PMID: 35163461 PMC8836193

[B40] Kordan B. Dancewicz K. Wróblewska A. Gabryś B. (2012). Intraspecific variation in alkaloid profile of four lupine species with implications for the pea aphid probing behaviour. Phytochem. Lett. 5, 71–77. doi: 10.1016/j.phytol.2011.10.003

[B41] Kordan B. Lahuta L. Dancewicz K. Sądej W. Gabryś B. (2011). Effect of lupin cyclitols on pea aphid probing behaviour. J. Plant Prot. Res. 51, 171–176. doi: 10.2478/v10045-011-0030-z

[B42] Kumar S. (2017). Plant secondary metabolites (PSMs) of Brassicaceae and their role in plant defense against insect herbivores–A review. J. Appl. Natural Sci. 9, 508–519. doi: 10.31018/jans.v9i1.1222

[B43] Kuzina V. Ekstrøm C. T. Andersen S. B. Nielsen J. K. Olsen C. E. Bak S. (2009). Identification of Defense Compounds in Barbarea vulgaris against the Herbivore Phyllotreta nemorum by an Ecometabolomic Approach. Plant Physiol. 151, 1977–1990. doi: 10.1104/pp.109.136952, PMID: 19819983 PMC2785962

[B44] Lee A. Hirsch A. M. (2006). Signals and responses: Choreographing the complex interaction between legumes and α-and β-rhizobia. Plant Signaling Behav. 1, 161–168. doi: 10.4161/psb.1.4.3143, PMID: 19521481 PMC2634022

[B45] Liu H. Brettell L. E. Qiu Z. Singh B. K. (2020). Microbiome-mediated stress resistance in plants. Trends Plant Sci. 25, 733–743. doi: 10.1016/j.tplants.2020.03.014, PMID: 32345569

[B46] Lu H. Yang P. Xu Y. Luo L. Zhu J. Cui N. . (2016). Performances of survival, feeding behavior and gene expression in aphids reveal their different fitness to host alteration. Sci. Rep. 6, 19344. doi: 10.1038/srep19344, PMID: 26758247 PMC4725932

[B47] Maag D. Erb M. Köllner T. G. Gershenzon J. (2015). Defensive weapons and defense signals in plants: some metabolites serve both roles. BioEssays 37, 167–174. doi: 10.1002/bies.201400124, PMID: 25389065

[B48] Martin B. Collar J. Tjallingii W. Fereres A. (1997). Intracellular ingestion and salivation by aphids may cause the acquisition and inoculation of non-persistently transmitted plant viruses. J. Gen. Virol. 78, 2701–2705. doi: 10.1099/0022-1317-78-10-2701, PMID: 9349493

[B49] Ng J. C. Perry K. L. (2004). Transmission of plant viruses by aphid vectors. Mol. Plant Pathol. 5, 505–511. doi: 10.1111/j.1364-3703.2004.00240.x, PMID: 20565624

[B50] O’Banion B. S. Jones P. Demetros A. A. Kelley B. R. Knoor L. H. Wagner A. S. . (2023). Plant myo-inositol transport influences bacterial colonization phenotypes. Curr. Biol. 33, 3111–3124. doi: 10.1016/j.cub.2023.06.057, PMID: 37419115

[B51] Olivon F. Elie N. Grelier G. Roussi F. Litaudon M. Touboul D. (2018). MetGem software for the generation of molecular networks based on the t-SNE algorithm. Analytical Chem. 90, 13900–13908. doi: 10.1021/acs.analchem.8b03099, PMID: 30335965

[B52] Osbourn A. E. (1996). Preformed Antimicrobial Compounds and Plant Defense against Fungal Attack. The Plant Cell 8, 1821–1831. doi: 10.1105/tpc.8.10.1821, PMID: 12239364 PMC161317

[B53] Pandharikar G. Gatti J.-L. Simon J.-C. Frendo P. Poirié M. (2020). Aphid infestation differently affects the defences of nitrate-fed and nitrogen-fixing Medicago truncatula and alters symbiotic nitrogen fixation. Proc. R. Soc. B 287, 20201493. doi: 10.1098/rspb.2020.1493, PMID: 32873201 PMC7542793

[B54] Pawłowska A. Stepczyńska M. (2022). Natural biocidal compounds of plant origin as biodegradable materials modifiers. J. Polymers Environ. 30, 1683–1708. doi: 10.1007/s10924-021-02315-y, PMID: 34720776 PMC8541817

[B55] Pitino M. Hogenhout S. A. (2013). Aphid protein effectors promote aphid colonization in a plant species-specific manner. Mol. Plant-Microbe Interact. 26, 130–139. doi: 10.1094/MPMI-07-12-0172-FI, PMID: 23035913

[B56] Pluskal T. Castillo S. Villar-Briones A. Orešič M. (2010). MZmine 2: modular framework for processing, visualizing, and analyzing mass spectrometry-based molecular profile data. BMC Bioinf. 11, 1–11. doi: 10.1186/1471-2105-11-395, PMID: 20650010 PMC2918584

[B57] Ponzio C. Papazian S. Albrectsen B. R. Dicke M. Gols R. (2017). Dual herbivore attack and herbivore density affect metabolic profiles of Brassica nigra leaves. Plant Cell Environ. 40, 1356–1367. doi: 10.1111/pce.12926, PMID: 28155236

[B58] Poole P. S. Ledermann R. (2022). Maintaining osmotic balance in legume nodules. J. Exp. Bot. 73, 8–10. doi: 10.1093/jxb/erab425, PMID: 34986228 PMC8730688

[B59] Pupel P. Szablińska-Piernik J. Lahuta L. B. (2019). Two-step d-ononitol epimerization pathway in Medicago truncatula. Plant J. 100, 237–250. doi: 10.1111/tpj.14439, PMID: 31215085

[B60] Ramachandran V. K. East A. K. Karunakaran R. Downie J. A. Poole P. S. (2011). Adaptation of Rhizobium leguminosarum to pea, alfalfa and sugar beet rhizospheres investigated by comparative transcriptomics. Genome Biol. 12, 1–12. doi: 10.1186/gb-2011-12-10-r106, PMID: 22018401 PMC3333776

[B61] Ranjbar Sistani N. Kaul H.-P. Desalegn G. Wienkoop S. (2017). Rhizobium impacts on seed productivity, quality, and protection of Pisum sativum upon disease stress caused by Didymella pinodes: phenotypic, proteomic, and metabolomic traits. Front. Plant Sci. 8, 1961. doi: 10.3389/fpls.2017.01961, PMID: 29204150 PMC5699443

[B62] Rodriguez P. A. Bos J. I. (2013). Toward understanding the role of aphid effectors in plant infestation. Mol. Plant-Microbe Interact. 26, 25–30. doi: 10.1094/MPMI-05-12-0119-FI, PMID: 23035915

[B63] Sanchez-Arcos C. Kai M. Svatoš A. Gershenzon J. Kunert G. (2019). Untargeted metabolomics approach reveals differences in host plant chemistry before and after infestation with different pea aphid host races. Front. Plant Sci. 10, 373118. doi: 10.3389/fpls.2019.00188, PMID: 30873192 PMC6403166

[B64] Shih P.-Y. Sugio A. Simon J.-C. (2023). Molecular mechanisms underlying host plant specificity in aphids. Annu. Rev. Entomol 68, 431–450. doi: 10.1146/annurev-ento-120220-020526, PMID: 36228134

[B65] Simon J.-C. Boutin S. Tsuchida T. Koga R. Le Gallic J.-F. Frantz A. . (2011). Facultative symbiont infections affect aphid reproduction. PloS One 6, e21831. doi: 10.1371/journal.pone.0021831, PMID: 21818272 PMC3144876

[B66] Smigielski L. Laubach E.-M. Pesch L. Glock J. M. L. Albrecht F. Slusarenko A. . (2019). Nodulation induces systemic resistance of Medicago truncatula and Pisum sativum against Erysiphe pisi and primes for powdery mildew-triggered salicylic acid accumulation. Mol. Plant-Microbe Interact. 32, 1243–1255. doi: 10.1094/MPMI-11-18-0304-R, PMID: 31025899

[B67] Stewart S. A. Hodge S. Bennett M. Mansfield J. W. Powell G. (2016). Aphid induction of phytohormones in Medicago truncatula is dependent upon time post-infestation, aphid density and the genotypes of both plant and insect. Arthropod-Plant Interact. 10, 41–53. doi: 10.1007/s11829-015-9406-8

[B68] Stochmal A. Simonet A. M. Macias F. A. Oleszek W. (2001). Alfalfa (Medicago sativa L.) flavonoids. 2. Tricin and chrysoeriol glycosides from aerial parts. J. Agric. Food Chem. 49, 5310–5314. doi: 10.1021/jf010600x, PMID: 11714321

[B69] Sulieman S. Schulze J. (2010). The efficiency of nitrogen fixation of the model legume Medicago truncatula (Jemalong A17) is low compared to Medicago sativa. J. Plant Physiol. 167, 683–692. doi: 10.1016/j.jplph.2009.12.016, PMID: 20207444

[B70] Szakiel A. Páczkowski C. Henry M. (2011). Influence of environmental abiotic factors on the content of saponins in plants. Phytochem. Rev. 10, 471–491. doi: 10.1007/s11101-010-9177-x

[B71] Taylor B. N. Ostrowsky L. R. (2019). Nitrogen-fixing and non-fixing trees differ in leaf chemistry and defence but not herbivory in a lowland Costa Rican rain forest. J. Trop. Ecol. 35, 270–279. doi: 10.1017/S0266467419000233

[B72] Tegeder M. (2014). Transporters involved in source to sink partitioning of amino acids and ureides: opportunities for crop improvement. J. Exp. Bot. 65, 1865–1878. doi: 10.1093/jxb/eru012, PMID: 24489071

[B73] Turetschek R. Desalegn G. Epple T. Kaul H.-P. Wienkoop S. (2017). Key metabolic traits of Pisum sativum maintain cell vitality during Didymella pinodes infection: cultivar resistance and the microsymbionts’ influence. J. Proteomics 169, 189–201. doi: 10.1016/j.jprot.2017.03.001, PMID: 28268116

[B74] War A. R. Paulraj M. G. Ahmad T. Buhroo A. A. Hussain B. Ignacimuthu S. . (2012). Mechanisms of plant defense against insect herbivores. Plant Signaling Behav. 7, 1306–1320. doi: 10.4161/psb.21663, PMID: 22895106 PMC3493419

[B75] Wu J. Baldwin I. T. (2010). New insights into plant responses to the attack from insect herbivores. Annu. Rev. Genet. 44, 1–24. doi: 10.1146/annurev-genet-102209-163500, PMID: 20649414

[B76] Yamada K. Mine A. (2024). Sugar coordinates plant defense signaling. Sci. Adv. 10, eadk4131. doi: 10.1126/sciadv.adk4131, PMID: 38266087 PMC10807812

[B77] Yan J. Lipka A. E. Schmelz E. A. Buckler E. S. Jander G. (2015). Accumulation of 5-hydroxynorvaline in maize (Zea mays) leaves is induced by insect feeding and abiotic stress. J. Exp. Bot. 66, 593–602. doi: 10.1093/jxb/eru385, PMID: 25271262 PMC4286406

[B78] Zuanazzi J. A. S. Clergeot P. H. Quirion J.-C. Husson H.-P. Kondorosi A. Ratet P. (1998). Production of Sinorhizobium meliloti nod gene activator and repressor flavonoids from Medicago sativa roots. Mol. Plant-Microbe Interact. 11, 784–794. doi: 10.1094/MPMI.1998.11.8.784

[B79] Züst T. Agrawal A. A. (2016). Mechanisms and evolution of plant resistance to aphids. Nat. Plants 2, 1–9. doi: 10.1038/nplants.2015.206, PMID: 27250753

