# Supplementary material for: Nitrogen-fixing symbiosis induces differential accumulation of Medicago truncatula leaf defence metabolites in response to pea aphid infestation
Source: Front Plant Sci. 2025 Nov 20;16:1670344. doi: 10.3389/fpls.2025.1670344 (PMC12675435; doi:10.3389/fpls.2025.1670344)
Supplement: Supplementary Data Sheet 1 — File containing qPCR Data (qPCR CT Data.xlsx). [file DataSheet1.docx]

**Supplementary Data for**

**Differential induction of *Medicago truncatula* defence metabolites in response to rhizobial symbiosis and pea aphid infestation**

Goodluck BENJAMIN^1^, Marie PACOUD^1^, Stéphanie BOUTET^2^, Gilles CLEMENT^2^, Renaud BROUQUISSE^1^, Jean-Luc GATTI^1^, Marylène POIRIÉ^1*^, Pierre FRENDO^1*^

1. Université Côte d’Azur, INRAE, CNRS, Institut Sophia Agrobiotech, 06903 Sophia Antipolis, France
2. Université Paris-Saclay, INRAE, AgroParisTech, Institut Jean-Pierre Bourgin for Plant Sciences (IJPB), 78000 Versailles, France

*Both authors supervised this work.

Correspondence: [pierre.frendo@univ-cotedazur.fr](mailto:pierre.frendo@univ-cotedazur.fr); [benjamingoodluckc@gmail.com](mailto:benjamingoodluckc@gmail.cometu.univ-cotedazur.fr)

**Supporting Information**

The metabolomics data that support the findings of this study are available in MassIVE server with the reference number MSV000095254; <http://doi.org/10.25345/C5CV4C35C>.

Datasets available as: Benjamin, Goodluck, 2024, "Supplementary Data for Differential induction of *Medicago truncatula* defence metabolites in response to rhizobial symbiosis and pea aphid infestation", <https://doi.org/10.57745/PCDG8H>, Recherche Data Gouv, V1, UNF:6:A0SbGGbxQocqa6JsmMuoMA== [fileUNF]

-Dataset S1: File containing qPCR Data (qPCR CT Data.xlsx)

-Dataset S2: File containing GCMS and LCMS metabolomics data (Metabolomics_data.xlsx)

-Dataset S3: File containing Significant accumulated metabolites analysed by Anova and Venn Analysis (Significant metabolites_Venn.xlsx)

**Fig. S1: Experimental setup.** Diagram showing timeline leading up to assay with key time points for bacteria inoculation and aphid infestation marked up. Each pot contained six plants and 4 pots were made for each condition, then 24 plants were used per condition for one biological replicate. Assay was performed using 4 biological replicates produced using the same timeline.

**Fig. S2: Expression of the housekeeping genes used for control in gene expression analysis**. Bar graph showing CT of housekeeping genes obtained by RT-qPCR analysis.

**Fig. S3: Experiment treatment classification.** Diagram showing the classification of studied treatments into 4 groups, NFS_Control, NFS_Amp, NI_Control (KNO_3_) and NI_Amp (KNO_3_). Amp means aphid infestation condition.

**Fig. S4:** **Abundance of quantified metabolites.** Graph of quantified metabolites from GC-MS analysis showing accuracy of abundance by comparing the mean obtained from the quality controls (QC, blue bars) and mean obtained from the samples (Samples, red bars).

**Table S1: List of genes and primer sequences used for RT-qPCR analysis.**

**Table S2: Gene expression analysis of NFS and NI plants.** Rescaled qPCR expression showing the level of gene induction upon aphid infestation in NFS and NI plants (NFS_Amp and NI_Amp, respectively) compared to their control (NFS and NI, respectively). ﻿Data are expressed as mean ± standard error (SE); t-test on all genes, P ≤ 0.05, significant.


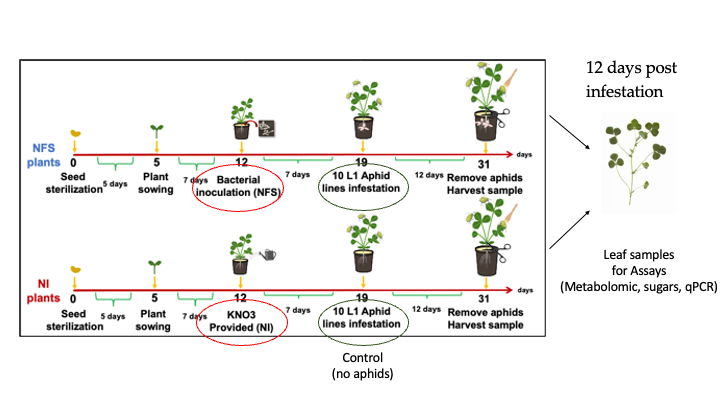


**Fig. S1: Experimental setup.** Diagram showing timeline leading up to assay with key time points for bacteria inoculation and aphid infestation marked up. Each pot contained six plants and 4 pots were made for each condition, then 24 plants were used per condition for one biological replicate. Assay was performed using 4 biological replicates produced using the same timeline.


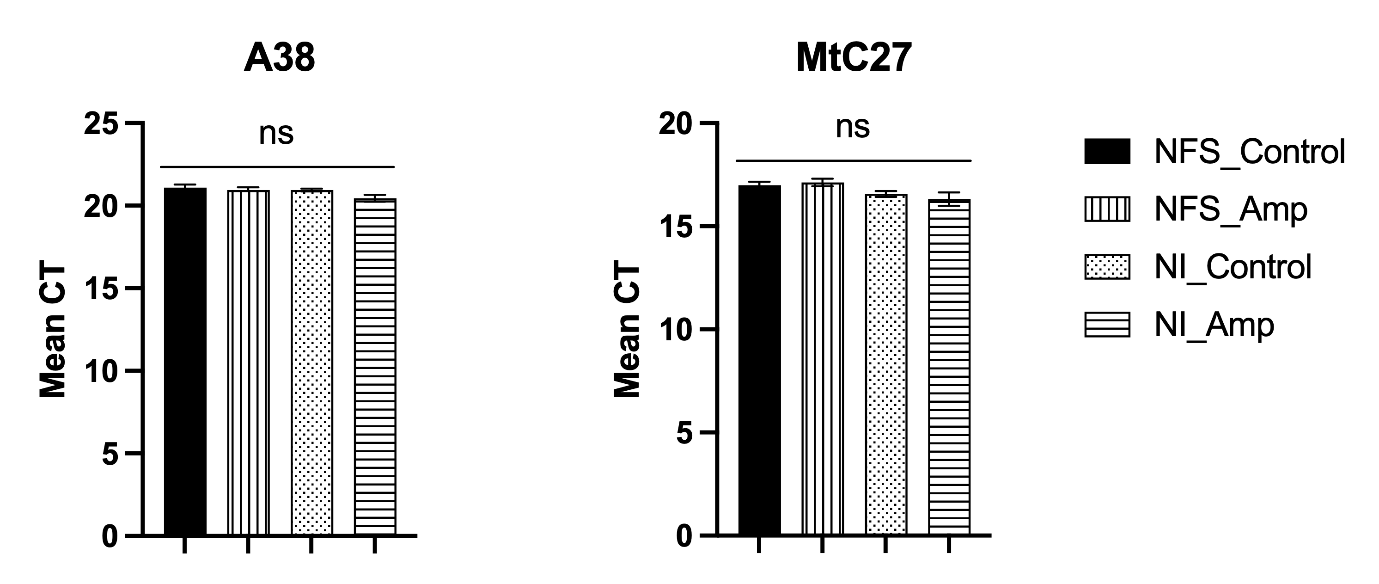


**Fig. S2: Expression of the housekeeping genes used for control in gene expression analysis**. Bar graph showing CT of housekeeping genes obtained by RT-qPCR analysis.


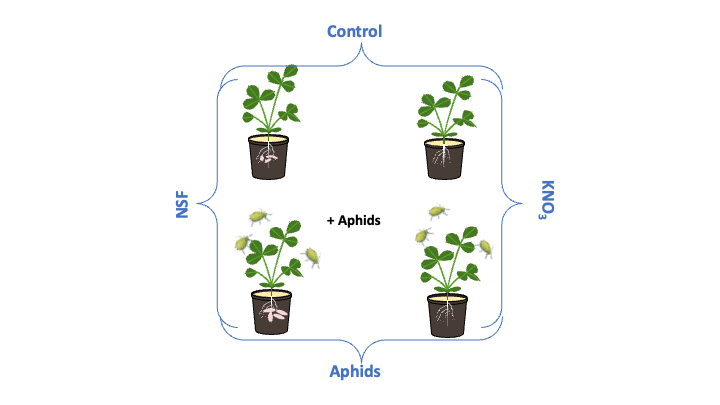


**Fig. S3: Experiment treatment classification.** Diagram showing the classification of studied treatments into 4 groups, NFS_Control, NFS_Amp, NI_Control (KNO_3_) and NI_Amp (KNO_3_). Amp means aphid infestation condition.


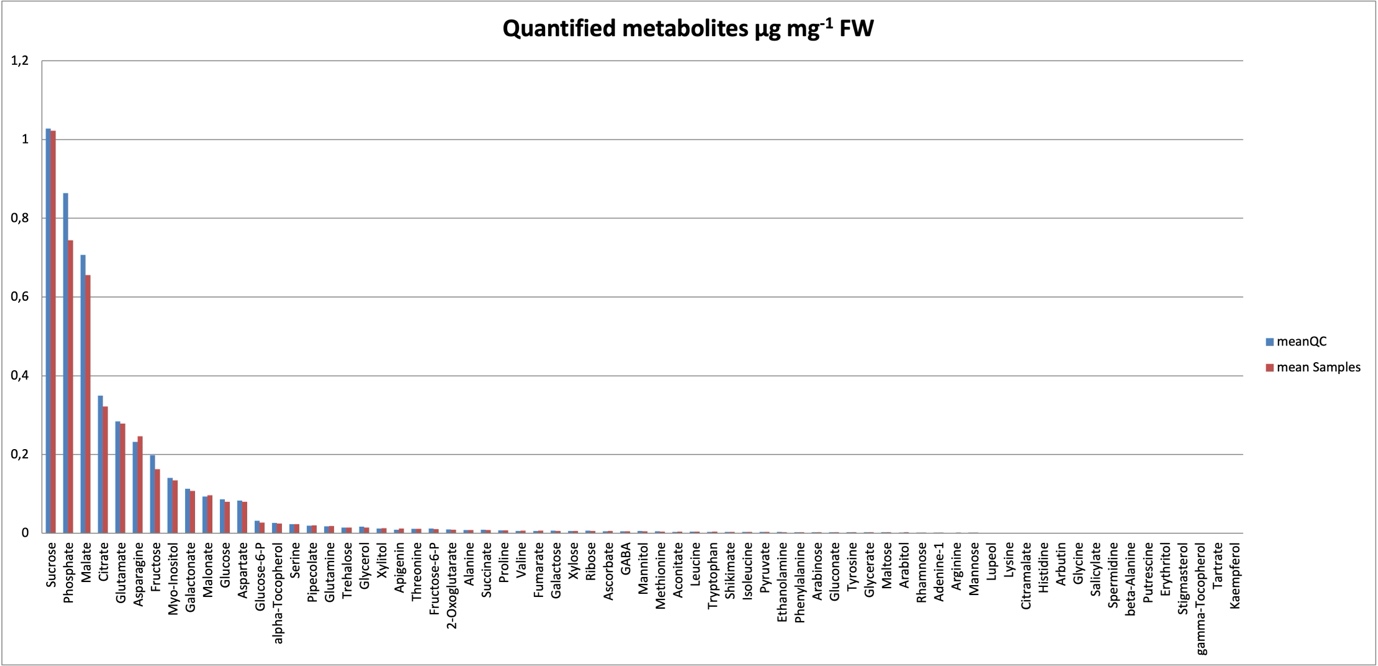


**Fig. S4:** **Abundance of quantified metabolites.** Graph of quantified metabolites from GC-MS analysis showing accuracy of abundance by comparing the mean obtained from the quality controls (QC, blue bars) and mean obtained from the samples (Samples, red bars).

**Table S1: List of genes and primer sequences used for RT-qPCR analysis.**

| **Description** | **Name** | **Genomic ID** | **Forward primer** | **Reverse primer** | **References** |
| --- | --- | --- | --- | --- | --- |
| Chalcone Isomerase | *CHI* | MtrunA17_Chr1g0213011 | CCTGAAAAGGAGGCTGCACT | ACAGCGCTTAAGATCAGGGG | This work |
| Flavanol Synthase/Flavanone 3-Hydroxylase | *FLS/F3H* | MtrunA17_Chr3g0092531 | CACAATTCACCAAAGAGATTGGGG | ATCCTCTGGCCTAGAAGGTGG | This work |
| Isoflavone 4'-O-methyltransferase | *HI4'O-MT* | MtrunA17_Chr4g0046341 | GCAACAGGGGAGAGTTTTTGG | AGACTCCAAACCCTCGAAAACG | This work |
| D-pinitol Dehydrogenase | *OEPB* | MtrunA17_Chr6g0480011 | GCACTCAGATGGGTGTACCA | GCACTCAGATGGGTGTACCA | This work |
| Phenylalanine Ammonia-lyase | *PAL* | MtrunA17_Chr1g0181091 | AGCGCTTATGTTAAAGCCGC | AGCGCTTATGTTAAAGCCGC | This work |
| Pterocarpan Synthase 1 | *PTS* | MtrunA17_Chr7g0259091 | AAGGAGATGCCATTGTGGAG | CCTATTCGTACTATAGGTGAGAGTG | This work |
| SAR Deficient 4 | *SARD4* | MtrunA17_Chr1g0202471 | CCCCAATTCGCCAACACTAC | CAGGGAAATGGGTCACGAGT | This work |
| Pathogenesis Related Protein-1 | *PR1* | MtrunA17_Chr2g0295371 | TTCGGGTTGGATGTGCTAAG | GGTTGAAGCTCAATGGCACT | Pandharikar *et al*., 2020 |
| Proteinase Inhibitor | *PI* | MtrunA17_Chr4g0014461 | TGTGGTGCAATTCTTTCAGG | ATTTTGGGGTGAGGTGTTGA | Pandharikar *et al*., 2020 |
| Housekeeping gene | *MtC27* | MtrunA17_Chr2g0295871 | TGAGGGAGCAACCAAATACC | GCGAAAACCAAGCTACCATC | Del Guidice *et al*., 2011 |
| Housekeeping gene | *a38* | MtrunA17_Chr4g0061551 | TCGTGGTGGTGGTTATCAAA | TTCAGACCTTCCCATTGACA | Del Guidice *et al*., 2011 |

**Table S2: Gene expression analysis of NFS and NI plants.**

| **Gene** | **Name** | **Pathway** | **Fold Increase from Control  (Mean** ± **SE)** | | ***p* value** | |
| --- | --- | --- | --- | --- | --- | --- |
|  |  |  | NFS_Amp/NFS | NI_Amp/NI | NFS_Amp/NFS | NI_Amp/NI |
| *PR1* | Pathogenesis Related Protein-1 | SA pathway | 3.5 ± 0.32 | 11.99 ± 1.25 | 0.05 | 0.02 |
| *PI* | Proteinase Inhibitor | JA pathway | 10.72 ± 0.26 | 4.73 ± 0.75 | 0.0006 | 0.002 |
| *PAL* | Phenylalanine Ammonia-lyase | Phenylpropanoid pathway | 1.40 ± 0.02 | 1.35 ± 0.14 | 0.06 | 0.31 |
| *SARD4* | SAR Deficient 4 | Pipecolate pathway | 1.16 ± 0.29 | 0.74 ± 0.09 | 0.73 | 0.88 |
| *OEPB* | D-pinitol Dehydrogenase | Pinitol pathway | 1.75 ± 0.01 | 1.96 ± 0.31 | 0.07 | 0.01 |
| *FLS/F3H* | Flavanol Synthase/Flavanone 3-Hydroxylase | Isoflavonoid pathway | 1.96 ± 0.05 | 2.5 ± 0.40 | 0.00002 | 0.01 |
| *HI4'O-MT* | Isoflavone 4'-O-methyltransferase | Isoflavonoid pathway | 4.32 ± 0.10 | 3.37 ± 0.53 | 0.001 | 0.02 |
| *CHI* | Chalcone Isomerase | Isoflavonoid pathway | 4.58 ± 0.11 | 5.83 ± 0.93 | 0.006 | 0.004 |
| *PTS* | Pterocarpan Synthase 1 | Isoflavonoid pathway | 53.35 ± 1.27 | 24.66 ± 3.91 | 0.0001 | 0.001 |

Rescaled qPCR expression showing the level of gene induction upon aphid infestation in NFS and NI plants (NFS_Amp and NI_Amp, respectively) compared to their control (NFS and NI, respectively). ﻿Data are expressed as mean ± standard error (SE); t-test on all genes, P ≤ 0.05, significant.
